# Screening germplasm and detecting QTLs for mesocotyl elongation trait in rice (*Oryza sativa* L.) by association mapping

**DOI:** 10.1186/s12863-023-01107-8

**Published:** 2023-02-15

**Authors:** Wisdom Mawuli Edzesi, Xiaojing Dang, Erbao Liu, William Kwame Nuako Bandoh, Patience Mansa Gakpetor, Daniel Aninagyei Ofori, Delin Hong

**Affiliations:** 1 State Key Laboratory of Crop Genetics and Germplasm Enhancement, Nanjing Agricultural University, Nanjing, 210095 China; 2 Council for Scientific and Industrial Research, Forestry Research Institute of Ghana, P. O. Box UP 63, KNUST, Fumesua, Kumasi, Ashanti Region Ghana; 3 Institute of Rice Research, Anhui Academy of Agricultural Sciences, Hefei, 230031 China; 4 College of Agriculture, Anhui Agricultural University, Hefei, 230036 China

**Keywords:** Dry direct seeding rice, Mesocotyl elongation length, Association mapping, Favorable marker allele, Simple sequence repeat

## Abstract

**Background:**

Rice is one of the most important food crops in the world and mainly cultivated in paddy field by transplanting seedlings. However, increasing water scarcity due to climate change, labor cost for transplanting, and competition from urbanization is making this traditional method of rice production unsustainable in the long term. In the present study, we mined favorable alleles for mesocotyl elongation length (MEL) by combining the phenotypic data of 543 rice accessions with genotypic data of 262 SSR markers through association mapping method.

**Results:**

Among the 543 rice accessions studied, we found 130 accessions could elongate mesocotyl length under dark germination condition. A marker-trait association analysis based on a mixed linear model revealed eleven SSR markers were associated with MEL trait with *p*-value less than 0.01. Among the 11 association loci, seven were novel. In total, 30 favorable marker alleles for MEL were mined, and RM265-140 bp showed the highest phenotypic effect value of 1.8 cm with Yuedao46 as the carrier accession. The long MEL group of rice accessions had higher seedling emergence rate than the short MEL group in the field. The correlation coefficient (*r* ^GCC−FSC^ = 0.485**) between growth chamber condition (GCC) and field soil condition (FSC) showed positive relationship and highly significant (*P* < 0.01) indicating that the result obtained in GCC could basically represent that obtained under FSC.

**Conclusion:**

Not every genotype of the rice possesses the ability to elongate its mesocotyl length under dark or deep sowing condition. Mesocotyl elongation length is a quantitative trait controlled by many gene loci, and can be improved by pyramiding favorable alleles dispersed at different loci in different germplasm into a single genotype.

**Supplementary Information:**

The online version contains supplementary material available at 10.1186/s12863-023-01107-8.

## Introduction

Rice (*Oryza sativa* L.) is one of the most common cereal crops grown in the world by transplanting seedlings into puddled soil. Traditional transplanted paddy rice is the major production system and nearly 95% of the paddy rice cultivation acreage is grown under such conditions with prolonged periods of flooding [1]. About 90% of the world’s rice is grown and produced (143 million ha of area with a production of 612 million tons of rough rice grain) in Asia [2]. However, in recent years, depleting water resources governed by climate change and labor shortage for rice seedling transplanting are threatening the sustainability and productivity of transplanted-flooded rice. Thus direct-seeded rice has been gradually accepted to be an optimal option for rice production [3]. There are three kinds of methods of rice direct seeding. They are dry direct seeding, wet direct seeding and water direct seeding. For water direct seeding, the rice seeds fall on the surface of the soil covered by 10–20 cm depth of water, and usually broadcast by airplane, like in California, USA. The seedling emergence rate under water direct seeding condition is related to the elongation length of coleoptile [4]. For dry direct seeding, there are three operational methods. They are (1) broadcasting of dry seeds on unpuddled soil after either zero tillage (such as Sri Lanka), (2) dibbled method in a well-prepared field (such as Malaysia and India) and (3) drilling of seeds in rows after conventional tillage (such as United States) [3]. Drill dry seeding is preferred over broadcasting in irrigated or favorable rainfed areas in both developed and developing countries because it simply allows line sowing and facilitates weed control between rows, saves time, and provides better crop establishment. For drill dry direct seeding, the rice seeds are buried under the soil, and the seedling emergence rate is related to the elongation length of mesocotyl.

Rice mesocotyl is an embryonic structure between the coleoptilar node and the basal part of the seedling, which elongates to push the shoot tip above the soil surface during germination [5]. The mesocotyl and coleoptile lengths are two crucial agronomic traits for direct seeding rice production because they can enhance seedling establishment [5–7]. Rice mesocotyl elongation only occurs in the dark environment and hence measurements of mesocotyl length can only be done when seed germination was completed under a dark environment, such as a growth chamber covered with black cloth or underground [8]. Rice mesocotyl elongation trait is genetically controlled and the elongation length is known to be controlled by quantitative trait loci (QTLs) and affected by many environmental factors [9]. To the best of our knowledge, up to now, a total of 18 QTLs for mesocotyl elongation length were identified on chromosomes 1(3), 2, 3(5), 5, 6(3), 7(2), 8, 11, 12 through linkage mappings [10–13]. A total of 30 association loci for mesocotyl elongation length were identified through genome-wide association study (GWAS). These association loci were located on chromosomes 1(4), 2, 3(2), 4(5), 5, 6(3), 7(6), 8, 9(4), 10, 11(2), [14–16]. Among all the loci identified above, one candidate gene, *OsGSK2*, was identified. *OsGSK2*, the key negative component in the Brassinosteroids signaling, is immediately adjacent to the lead single-nucleotide polymorphism (SNP) in a major associated locus on chromosome 5 (*P* = 3 × 10^–8^) and is within an 87 bp distance from the lead SNP of this associated locus suggesting that *OsGSK2* is a strong candidate gene in this associated locus [17]. Another gene identified to negatively regulate rice seedling mesocotyl elongation is *OsPAO5* which helps in breeding direct-seeding rice by targeted mutagenesis. Mutants of *OsPAO5* synthesize more ethylene and produce lower amounts of H_2_O_2_, resulting in a longer mesocotyl, faster seedling emergence, and higher yield potential [18]. According to the result of GWAS from other authors, eight and five high-confidence candidate genes known to be related to mesocotyl elongation were identified on chromosomes 1(2), 3,4,6,7(2),9 and 1, 3, 5, 9, 12, respectively were involved in the biological metabolism of phytohormones, cell elongation and division [19, 20].

Environmental factors influencing rice MEL included seeding depth, changes in aeration, light and temperature [21]. Rice mesocotyl elongation length was also regulated by strigolactones and cytokinins during germination of rice seeds in darkness [22–26]. And brassinosteroids, ethephon and gibberellic acid were also involved in the elongation process [27, 28]. Therefore, by adjusting seedling growth conditions, it is possible to alter patterns of mesocotyl elongation.

The objectives of this study were to (1) investigate the degree of variation of MEL within the selected natural population; (2) detect QTLs within the population using GWAS method; (3) mine favorable alleles and predict superior combinations for improving the MEL.

## Materials and methods

### Plant materials

Among the 543 rice genotypes used in this study, 121 accessions come from Vietnam (17°N – 23°N), 11 from Japan (20°N – 54°N) and the rest from China. The names of genotypes, their sources of origin are listed in Additional file 1: Table S1.

### Seed multiplication on the paddy field

All the 543 rice accessions were planted out in a paddy field at the Jiangpu Experimental Station, Nanjing Agricultural University, China (118.62°E, 32.07°N) from early May to November in 2016 using a randomized complete block design with three replications in an eight- column × five-row area. For all accessions, seedlings aged about 30 days were transplanted onto the paddy field at a spacing of 20 cm between rows and 17 cm between each individual. Standard agronomic management practices were adopted during the whole growth period after transplanting. The fresh seeds were harvested at 30 – 40 days after flowering and dried naturally in the sun.

### MEL evaluation in growth chamber covered with black cloth

**Fig. 1:**
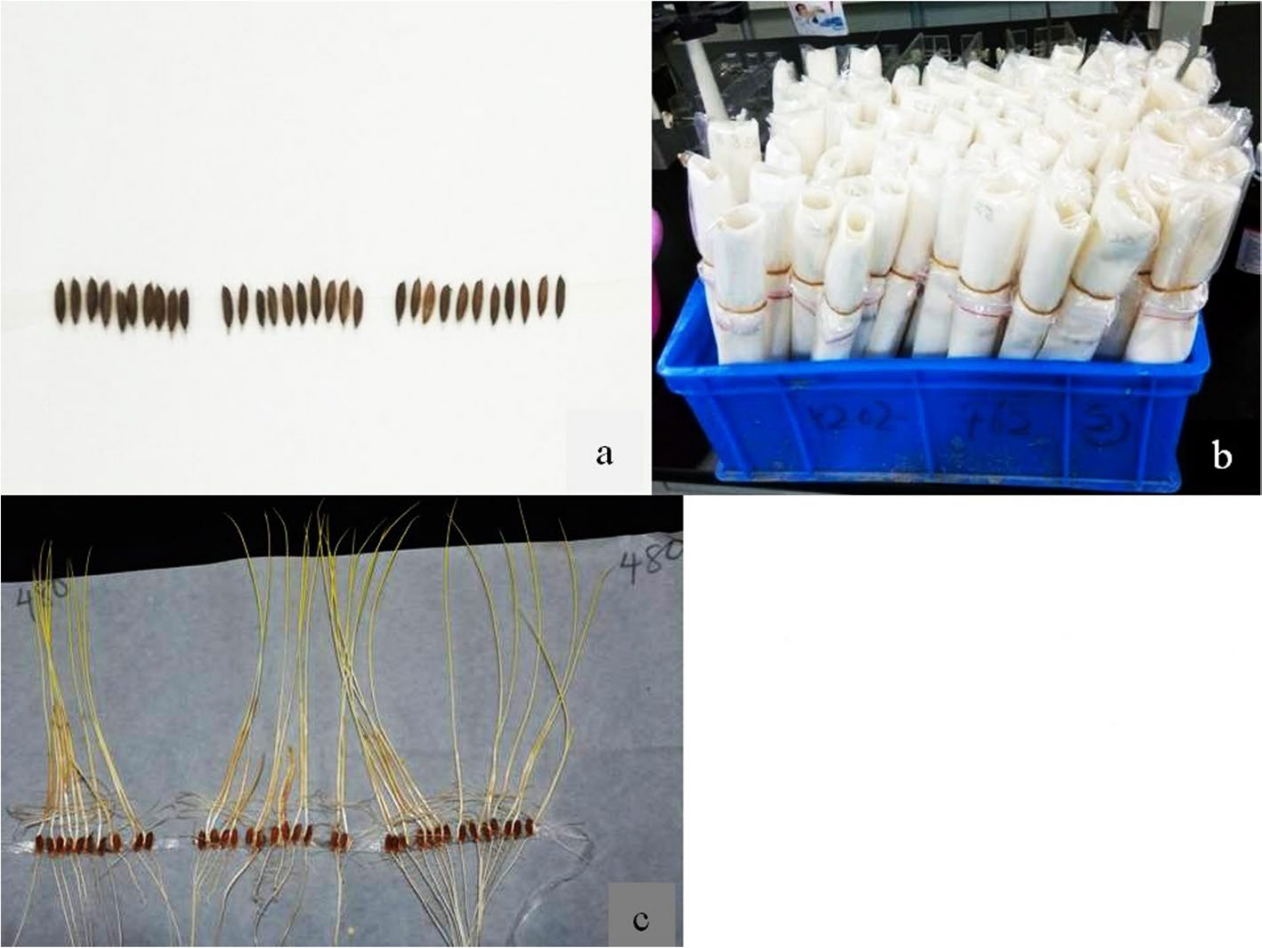
Preparation of rice materials for screening in the growth chamber. **a** Seeds are arranged on absorbent filter paper. **b** Absorbent paper rolled over water to make it wet placed in a plastic box. **c** displayed seeds after 10 days in the growth chamber at a controlled temperature of 30º C

For each accession, 30 plump, dry and disease-free fresh seeds were treated in an oven at 45˚C for 3 days to eliminate dormancy. The seeds were then wrapped in 20 × 20 cm wet absorbent filter paper, placed in a plastic box (60 cm × 30 cm × 30 cm) vertically and cultivated at 30˚C in total darkness in a growth chamber (Fig. 1). During germination, tap water was sprinkled to keep the filter paper moist. After 10 days, the mesocotyl elongation length of each etiolated seedling was measured by a ruler. The evaluation experiment was replicated three times biologically for each accession.

### MEL evaluation on the field soil condition

In order to clarify the relationship between the MEL observed in the growth chamber and the MEL under field soil sowing conditions for the same accession, we selected 30 accessions with long MEL [longer than 0.59 cm] observed in dark growth chamber to sow the seeds in the field soil at 5 cm depth, and 30 accessions with short MEL [longer than 00 mm, but shorter than 0.60 cm] observed in dark growth chamber to sow the seeds in the field soil at 2 cm depths. The sowing depths of 2 cm and 5 cm represent shallow and deep seeding practices respectively.

Thirty clean healthy seeds of each accession were sterilized by soaking in 0.6% sodium hypochlorite (NaClO) solution for 15 min, and later washed thoroughly with tap water. It was then soaked in tap water for 48 h to pre-germinate at room temperature. Germinated seeds were sown on the surface of field soil framed in rectangular constructed wooden boxes with 90 cm (length) × 20 cm (width) × 30 cm (high), then covered with cohesive, continuously moistened fine soil at 2-cm and 5-cm depths, cultivated on the field condition (that means under natural condition, no control for light length and intensity, day and night temperature, and so on). After sowing, the numbers of emerged seedlings were recorded until total seedling emergence of all accessions remained stable. Numbers of emerged seedlings from the soil surface were counted daily up to 12 days when seedling emergence was complete. Then, the MELs were measured with a ruler after being carefully cleaned from soil and this experiment was performed with three replications.

### SSR marker genotyping

Genomic DNA was extracted from the leaf tissue of one single plant in each plot (the plants within a plot were homogeneity) according to the methods described by Murray and Thompson (1980) [29]. According to the published rice molecular map and microsatellite database of Temnykh et al*.* (2000) [30] and McCouch et al*.* (2002) [31], 262 SSRs scattered on 12 chromosomes were selected. The primers were synthesized by Shanghai Generay Biotech Co. Ltd., Shanghai, China. Each 10 μl PCR reaction solution contained 10 mM Tris–HCl (pH 9.0), 50 mM KCl, 0.1% Triton X-100, 1.5 mM MgCl2, 0.5 nM dNTPs, 0.14 pM forward primers, 0.14 pM reverse primers, 0.5 units Taq polymerase, and 20 ng genomic DNA. The DNA amplification was performed using a PTC-100TM Peltier Thermal Cycler (MJ ResearchTM Incorporated, USA) under the following conditions: 1) denaturation at 94 °C for 5 min; 2) 34 cycles of denaturation at 94 °C for 0.5 min, annealing at 55–63 °C for 1 min, and extension at 72 °C for 1 min; and 3) final extension at 72 °C for 10 min. The PCR products were run on an 8% polyacrylamide gel at 150 V for 1 h and visualized using silver staining. One pair of SSR markers detected one locus, and each polymorphic band at the same marker locus in the population was recorded as one allele. After screening the polyacrylamide gel electrophoresis (PAGE) products, the molecular weight of each band was calculated by the software Quantity One. The SSR marker genotyping data of the 543 accessions with 262 SSR markers were listed on Additional file 2: Table S2.

### Data analysis

The mean, standard deviation (SD), range, kurtosis and skewness and broad-sense heritability (*H*^*2*^_*B*_) were calculated using Excel 2007 and Minitab 17 respectively. The broad-sense heritability (*H*^*2*^_*B*_) was computed by an ANOVA using the following formula:

$${{H}^{2}}_{B}={{\sigma }^{2}}_{g}/\left({{\sigma }^{2}}_{g}+{{\sigma }^{2}}_{e}/n\right)$$

where* σ*^*2*^_*g*_ is genetic variance, *σ*^*2*^_*e*_ is error variance, and *n* is the number of replications.

Correlation coefficients were calculated between MELs under growth chamber and MELs under FSC for the same materials to elucidate the relationship between the two results. The number of alleles per locus, gene diversity per locus and polymorphism information content (PIC) per locus were determined using PowerMarker version 3.25. Nei’s genetic distance was used for the unrooted phylogeny reconstruction [32]. The optimum number of subpopulations (K) was selected after five independent runs of a burn-in of 50,000 iterations followed by 100,000 iterations for each value of K (from 2 to 10) using Structure software version 2.3. Linkage Disequilibrium (LD) level was estimated by the D' value between all pairs of SSRs with 1,000 permutations and calculated using TASSEL 3.0 software [33–35].

The associations between MEL and SSR markers were analyzed using a mixed linear model (MLM) in TASSEL 3.0 [34, 35]. In this study, alleles with positive effects (*P* < 0.05) are considered favorable alleles for MEL measured and alleles with frequencies of less than 5% in the population were regarded as rare alleles and treated as missing data. The following formula was used to calculate the positive (negative) average allele effect (AAE) of each locus:

$$AAE=\sum ai/ni$$

where *a*_*i*_ is the positive (negative) allelic phenotypic effect values of locus *i*, and *n*_*i*_ is the number of positive (negative) alleles within locus* i*.

## Results

### Analysis of phenotypic data obtained from the dark growth chamber

**Fig. 2:**
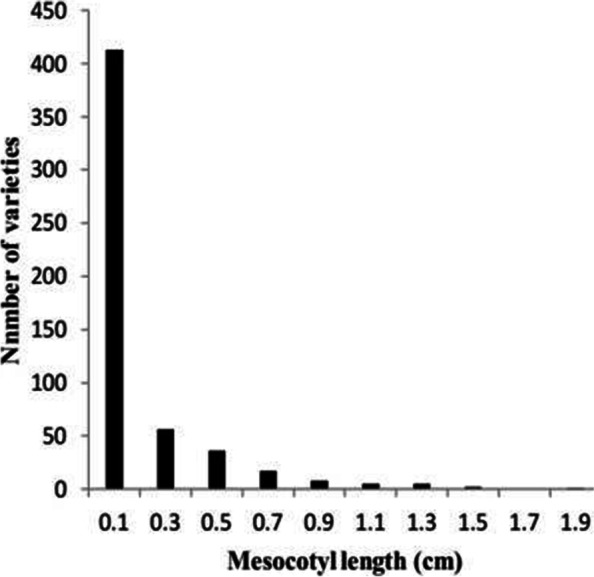
Histogram of grouped frequency distribution for mesocotyl elongation lengths

**Fig. 3:**
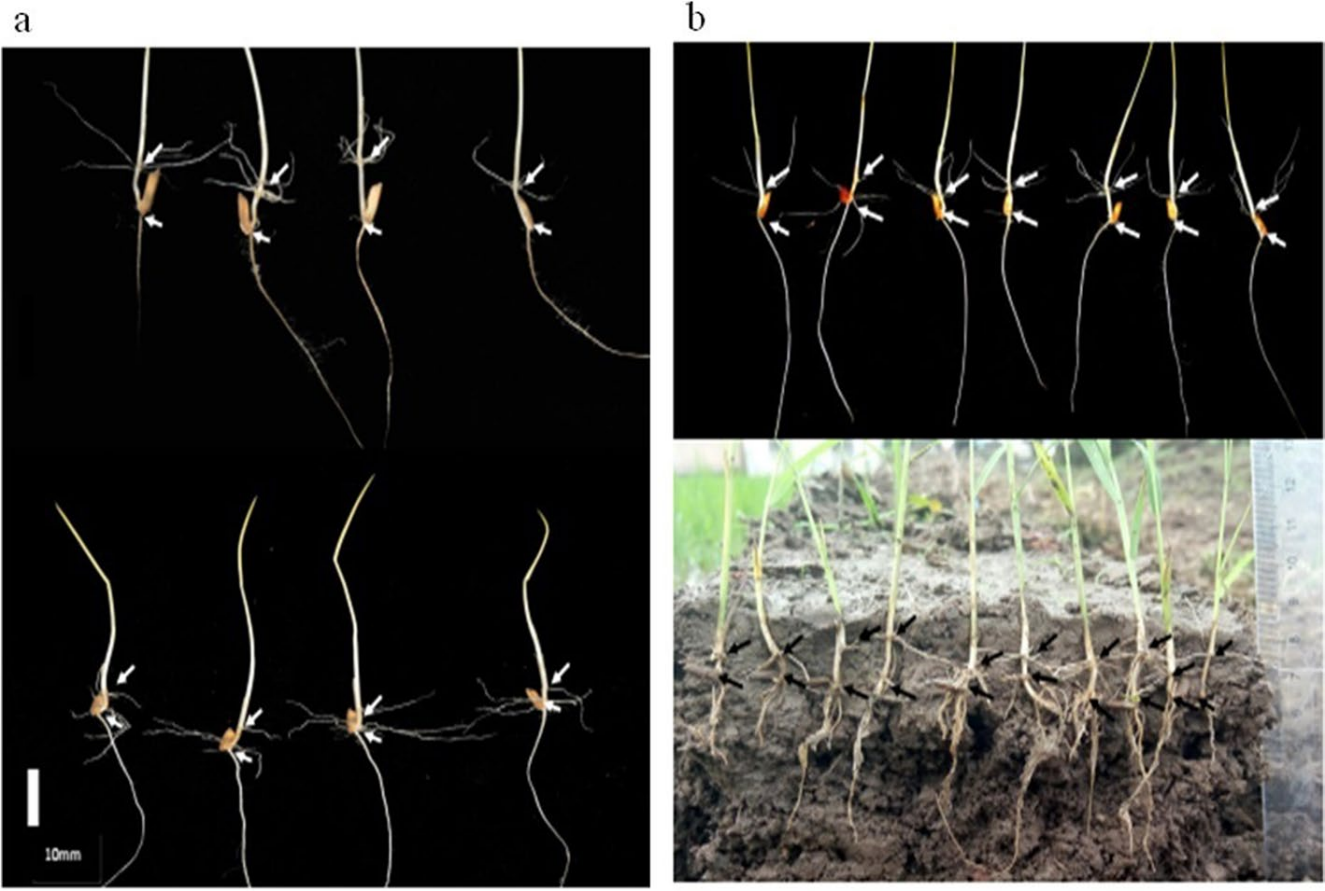
Examples of different mesocotyl elongation lengths observed in darked growth chamber (**a, upper part of b**) and in the soil conditions (**lower part of b**)

The phenotypic data of MEL followed a skewed distribution based on the values of skewness and kurtosis statistics. The mean of MEL over the 543 accessions was 0.11 cm, the range of MEL over 543 accessions was from 0.00 to 1.88 cm, kurtosis and skewness were 11.95 and 3.13 respectively, and it was discovered there were 413 accessions with no MEL (shorter than 0.15 cm, Fig. 2). Among the 130 accessions with MEL, Yuedao46 recorded the highest MEL of 1.88 cm, and Yuedao40 and Yuesdao64 both recorded the lowest mesocotyl elongation length of 0.15 cm (Table 1). Figure 3 showed graphical display of MELs of some accessions from both GCC and in the FSC for MEL measurement.

**Table 1:** Mesocotyl elongation lengths of 130 varieties under growth chamber for 10 days

Variety Name	MEL/cm	Origin	Sub-population
Yuedao46	1.88	Vietnam	5
Yuedao59	1.50	Vietnam	5
Yuedao20	1.14	Vietnam	5
Yuedao50	1.12	Vietnam	5
Yuedao70	1.05	Vietnam	5
Yuedao36	1.03	Vietnam	5
Yuedao30	0.92	Vietnam	5
Yuedao44	0.77	Vietnam	5
Yuedao109	0.72	Vietnam	5
Yuedao97	0.72	Vietnam	5
Yuedao48	0.70	Vietnam	5
Yuedao67	0.67	Vietnam	5
Yuedao34	0.64	Vietnam	5
Yuedao93	0.60	Vietnam	5
Yuedao31	0.60	Vietnam	5
Yuedao22	0.59	Vietnam	5
Yuedao38	0.54	Vietnam	5
Yuedao25	0.53	Vietnam	5
Yuedao32	0.53	Vietnam	5
Yuedao23	0.50	Vietnam	5
Yuedao43	0.48	Vietnam	5
Yuedao55	0.45	Vietnam	5
Yuedao56	0.41	Vietnam	5
Yuedao37	0.40	Vietnam	5
Yuedao33	0.39	Vietnam	5
Yuedao96	0.39	Vietnam	5
Yuedao42	0.39	Vietnam	5
Yuedao26	0.37	Vietnam	5
Yuedao110	0.36	Vietnam	5
Yuedao54	0.36	Vietnam	5
Yuedao94	0.35	Vietnam	5
Yuedao51	0.35	Vietnam	5
Yuedao107	0.34	Vietnam	5
Yuedao2	0.34	Vietnam	5
Yuedao65	0.34	Vietnam	5
Yuedao47	0.33	Vietnam	5
Yuedao72	0.32	Vietnam	5
Yuedao24	0.31	Vietnam	5
Yuedao77	0.30	Vietnam	5
Yuedao45	0.28	Vietnam	5
Yuedao99	0.27	Vietnam	5
Yuedao89	0.27	Vietnam	5
Yuedao28	0.27	Vietnam	5
Yuedao61	0.26	Vietnam	5
Yuedao60	0.25	Vietnam	5
Yuedao21	0.25	Vietnam	5
Yuedao52	0.25	Vietnam	5
Yuedao108	0.24	Vietnam	5
Yuedao86	0.23	Vietnam	5
Yuedao53	0.23	Vietnam	5
Yuedao87	0.18	Vietnam	5
Yuedao39	0.17	Vietnam	5
Yuedao64	0.15	Vietnam	5
Yuedao40	0.15	Vietnam	5
Fuyu3	1.16	Yuexi, Anhui	4
Heijing8	1.08	Haerbin, Heilongjiang	4
Beidao4	0.81	Haerbin, Heilongjiang	4
Yuedao5	0.74	Vietnam	4
Datougui	0.72	Changshu, Jiangsu	4
Mudanjiang28	0.55	Mudanjiang, Heilongjiang	4
Yuedao3	0.42	Vietnam	4
Yuedao7	0.35	Vietnam	4
C418	0.35	Shenyang, Liaoning	4
Wanyangdao	0.33	Wuxian, Jiangsu	4
Aidazhong	0.33	Wujiang, Jiangsu	4
Zaonuodao	0.27	Wujiang, Jiangsu	4
Mudanjiang27	0.25	Mudanjiang, Heilongjiang	4
Yuedao9	0.25	Vietnam	4
Yuedao10	0.25	Vietnam	4
Nannongjing3786	0.23	Nanjing, Jiangsu	4
Yuedao115	0.17	Vietnam	4
Yuedao116	0.16	Vietnam	4
Xiaofenghuang	1.23	Wuxian, Jiangsu	3
Xiangqing	1.19	Chongming, Shanghai	3
Jiuxiaozhong	0.82	Wujiang, Jiangsu	3
Zhen6	0.63	Zhengzhou, Henan	3
Katena	0.56	Wujiang, Jiangsu	3
Xishihuang	0.56	Wuxian, Jiangsu	3
Chuyanghan32	0.40	Wuxian, Jiangsu	3
Zhendao10	0.37	Zhenjiang, Jiangsu	3
Qianjindao	0.33	Wujiang, Jiangsu	3
Cungu	0.32	Wujiang, Jiangsu	3
Qijiangqing	0.31	Kunshan, Jiangsu	3
Chiguwandao	0.29	Wujiang, Jiangsu	3
Jianongnuo2	0.29	Wujiang, Jiangsu	3
Wandao68	0.26	Hefei, Anhui	3
Louhanbai	0.26	Kunshan, Jiangsu	3
Zhengdao18	0.26	Zhenzhou, Henan	3
Yishixing	0.25	Changshu, Jiangsu	3
Lujingqing	0.25	Wujiang, Jiangsu	3
Huaidao11	0.24	Huaian, Jiangsu	3
Wumangyedao	0.21	Changshu, Jiangsu	3
Libanyi	0.20	Wuxian, Jiangsu	3
Yanjing8	0.18	Yancheng, Jiangsu	3
Zijing	0.16	Nanjing, Jiangsu	3
Jindao12	0.16	Dongli, Tianjin	3
Zhoujiazhong	0.15	Wujiang, Jiangsu	3
Huangsandannuo	0.61	Wuxi, Jiangsu	2
Xianhui429	0.61	Nanjing, Jiangsu	2
Shengtangdao	0.56	Changshu, Jiangsu	2
Yangdao6	0.37	Vietnam	2
Wuyujing3	0.35	Wujin, Jiangsu	2
Kongqueqing	0.31	Kunshan, Jiangsu	2
Manyedao	0.30	Kunshan, Jiangsu	2
Jia159	0.28	Jiaxing, Zhejiang	2
Diantun502xuanzao	0.26	Kunming, Yunnan	2
Huaidao9	0.26	Huaian, Jiangsu	2
Nannongjing62401	0.26	Nanjing, Jiangsu	2
Ebusinuodao	0.22	Wuxi, Jiangsu	2
Yandao6	0.21	Yancheng, Jiangsu	2
Wumangzaodao	0.20	Changshu, Jiangsu	2
Xiushui79	0.17	Jiaxing, Zhejiang	2
Cuyingwanyangdao	0.17	Wuxi, Jiangsu	2
Si4386	1.36	Sihong, Jiangsu	1
Xiangchuanwuxinbaimi	1.09	Haerbin, Heilongjiang	1
Kasala	0.79	Haerbin, Heilongjiang	1
Nongxiang25	0.62	Changsha, Hunan	1
Zidao	0.36	Nanjing, Jiangsu	1
Si4263	0.34	Sihong, Jiangsu	1
Ningjinghui290	0.29	Nanjing, Jiangsu	1
Ningjinghui296	0.29	Nanjing, Jiangsu	1
Ningjinghui293	0.27	Nanjing, Jiangsu	1
Ningjinghui145	0.26	Nanjing, Jiangsu	1
Nongxiang26	0.26	Changsha, Hunan	1
Si4081	0.25	Sihong, Jiangsu	1
Yuzhenxiang	0.25	Changsha, Hunan	1
Ningjinghui286	0.25	Nanjing, Jiangsu	1
Fengyouwan8	0.25	Changsha, Hunan	1
Nongxiang21	0.24	Changsha, Hunan	1
Ningjinghui260	0.19	Nanjing, Jiangsu	1

Among the 130 accessions that show MEL under dark GCC, 54 accessions (occupying 41.6%) were from Vietnam, 25 accessions (19.2%) were from the eastern part of China), and 16 accessions (12.3%) were from northern China) (Table 1).

### Genetic diversity of SSR markers

**Fig. 4:**
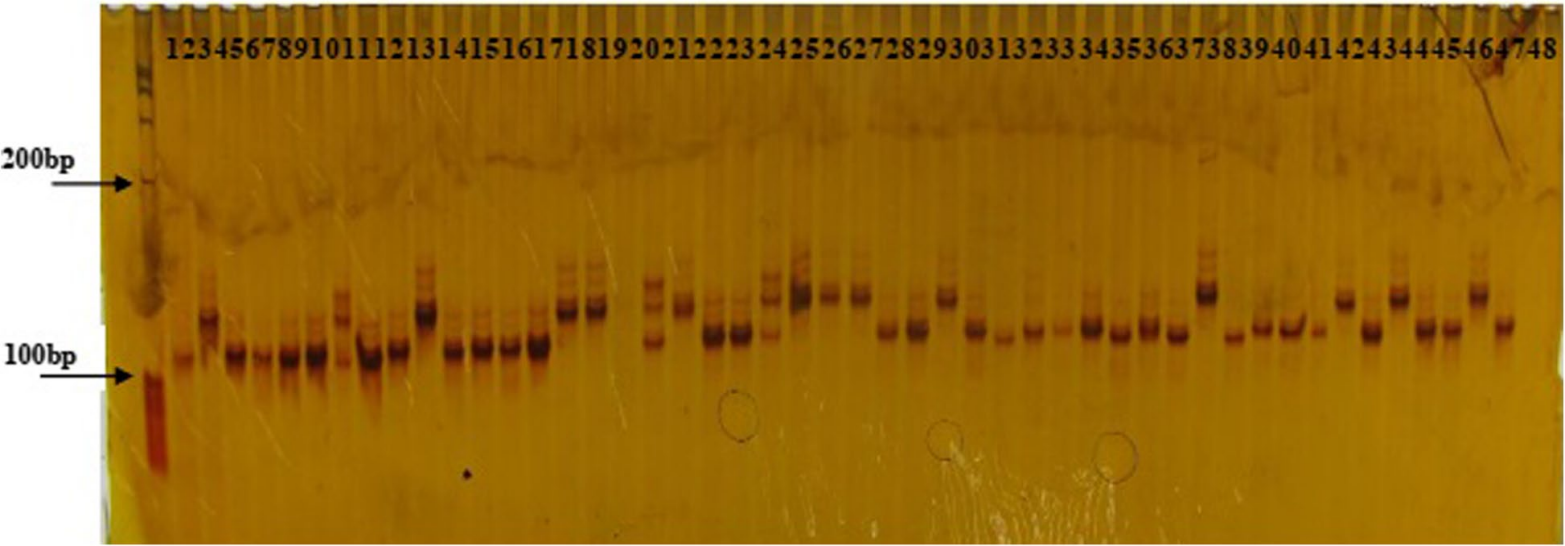
SSR profile amplified by RM471, using genome DNA as template.The first column from left of the gel is DNA size marker. Digits from 1 to 48 are codes of accessions used in this study

All 262 SSR markers were polymorphic, and they produced a total of 2,649 alleles among the 543 assayed accessions. The average number of alleles per locus was 10.107, with values ranging from 2 (RM437 on Chr 5 and RM206 on Chr 11) to 25 (RM7545 on Chr 10) (Fig. 4, Additional file 3: Table S3). The average genetic diversity over all SSR loci was 0.734, with values ranging from 0.0802 (RM206 on Chr 11) to 0.9429 (RM7545 on Chr 10) (Additional file 3: Table S3). The mean polymorphism information content (PIC) value was 0.706, with values ranging from 0.077 (RM206 on Chr 11) to 0.940 (RM7545 on Chr 10) and a major distribution between 0.456 and 0.940 (Additional file 3: Table S3). Two hundred and twenty-nine markers (87.4%) were highly informative (PIC > 0.5), 27 (10.3%) were moderately informative (0.5 > PIC > 0.25) and 6 (2.3%) were slightly informative (PIC < 0.25).

### Population structure and genetic relatedness

**Fig. 5:**
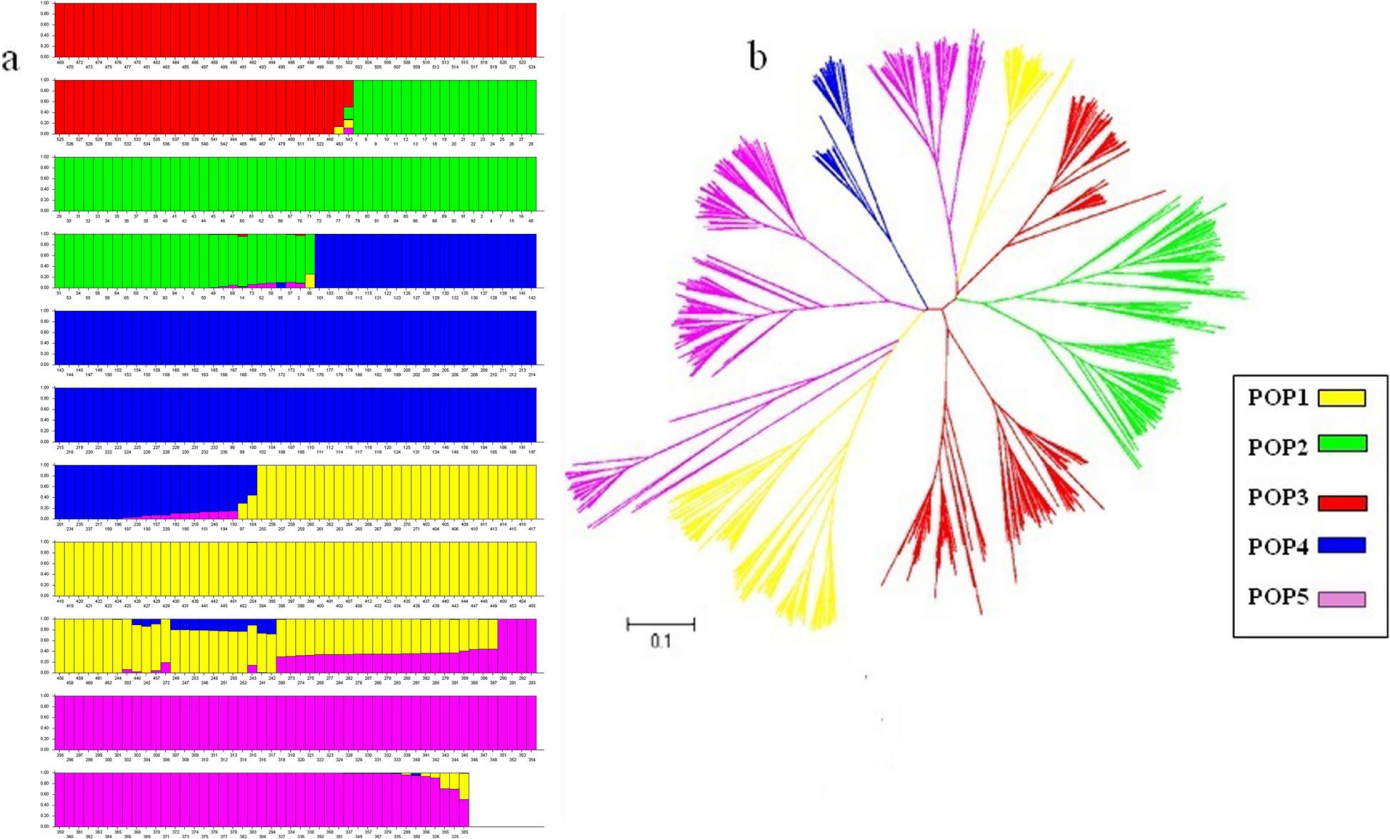
Structure analysis of 543 rice accessions using a: STRUCTURE; Posterior probability of each rice variety of 543 rice accessions belonging to 5 subpopulation. **a** Each accession is represented by a vertical bar. The colored subsection within each vertical bar indicate membership coefficient (Q) of the accession to different clusters. Identified subpopulation are POP1 (yellow), POP2 (gree), POP3 (red), POP4 (blue), POP5 (purple). **b** A neighbor-joining tree for 543 rice accessions based on Nei’s genetic dostance

An analysis of the model-based population structure provided evidence of a significant population stratification structure in the 543 rice accessions and identified the highest likelihood value at K = 5 for all five replicates (five runs for each K) Therefore, the entire population can be divided into 5 sub-populations. The posterior probability value of each accession belonging to the five subpopulations is shown in Fig. 5a.

The population structure data based on the Q matrix for each accession are summarized in Additional file 1: Table S1. A neighbor-joining tree of the 543 accessions was constructed based on Nei’s genetic distance (Fig. 5b), and the results were consistent with the results from the STRUCTURE analysis. For instance, accessions from northeastern China and Japan clustered in POP2 while the accessions in POP3 represented landraces from eastern China (Taihu Lake valley); accessions in POP4 were mainly from northern China (northern Jiangsu, Anhui, and Shandong provinces and Tianjin City) and POP 5 were mainly from Vietnam. Among the 130 accessions that show MEL under GCC, 54 accessions were from Vietnam (occupying 41.6%), 25 accessions were from the eastern part of China (19.2%), and 16 accessions come from northern China (12.3%) (Table 2).

**Table 2:** Comparison of *D*ʹ values for pair-wise SSR loci in five subpopulations

Cluster	No. of LD	Ratio^b^ (%)	Frequency of D′ value (*P* < 0.05)					Means of D^c^′
	**locus pairs**^**a**^							
			**0–0.20**	**0.21–0.40**	**0.41–0.60**	**0.61–0.80**	**0.81–1.0**	
POP1	825	23.2	4	74	284	324	139	0.625
POP2	994	27.9	0	117	308	369	200	0.633
POP3	1128	31.7	2	110	302	434	280	0.66
POP4	32	0.9	0	0	8	14	10	0.718
POP5	583	1.6	7	104	181	118	173	0.628

^a^LD means linkage disequilibrium

^b^Ratio between the number of intrachromosomal significant LD locus pairs and total number of significant LD locus pairs

^c^*D*ʹ means standardized disequilibrium coefficients

**Fig. 6:**
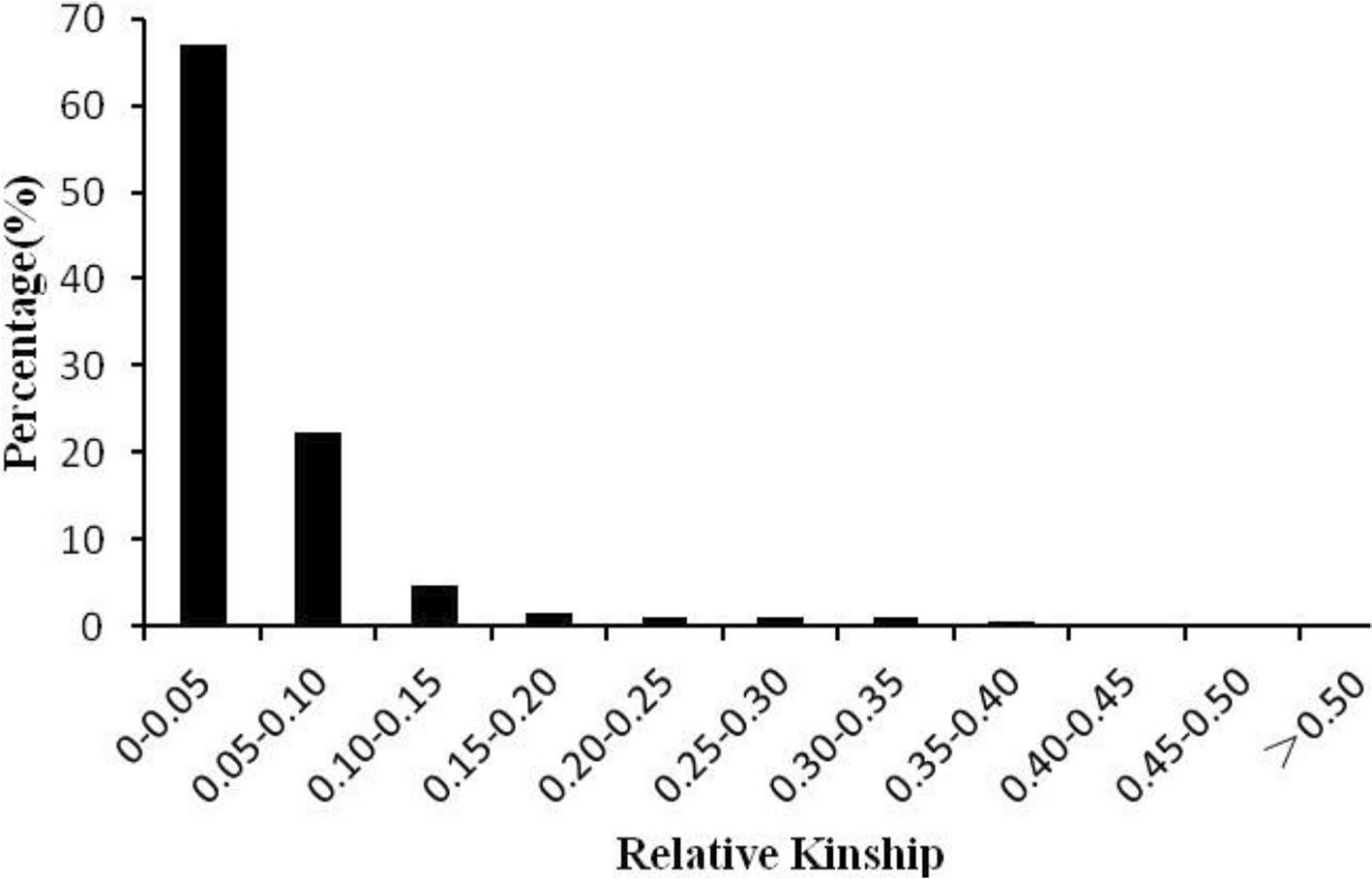
Histogram of distribution of pair-wise relative kinship coefficient groups among 543 rice accession based on 262 SSR markers

Genetic relatedness analysis showed that the accessions in this study were distantly, with greater than 60% of the kinship coefficient values at less than 0.05, 22.4% ranging from 0.05–0.10 and the remaining 10.64% showing various degrees of genetic relatedness (Fig. 6). This result only goes to imply how weak relatedness exists between the pairwise rice accessions. Based on the results of the relatedness analysis, a K matrix was constructed for the association analysis.

### Linkage disequilibrium analysis

Linkage disequilibrium (LD) is a population-based parameter that describes the degree to which an allele of one genetic variant is inherited or correlated with an allele of a nearby genetic variant within a given population [36]. Population geneticists calculate LD to assess population structure and population history [37] and LD analysis can be employed to detect natural selection and estimate allelic age [38]. Among the 5 subpopulations, the lowest significant pair-wise loci and percentage of significant pair-wise loci in LD was found in POP4 (32, 0.9% respectively), and the highest significant pair-wise loci was found in POP3 (1128, 31.7% respectively) (Table 2). POP4 had the highest average of D′ (0.718) among the 5 subpopulations while POP1 had the lowest average of D′ (0.625) among the 5 subpopulations (Table 2), suggesting that POP1 accessions in this subpopulation might be subjected to some sort of artificial selection.

### Phenotypic analysis of MEL observed on the field soil

#### Accessions at 5-cm depth (Long MEL)

A MEL comparison of accessions classified under Long MEL group that were sowed at 5-cm depth compared to the GCC. These 30 accessions were selected from the growth chamber condition based on their performance of exhibiting long MEL. Comparing the accessions performance under these two environments, Yuedao46 had the highest MEL (1.88 cm) among the 30 accessions under GCC followed by Yueado59 but on the field soil condition, Heijing8 had the highest MEL followed by Kasala variety of 3.1 cm, and 2.60 respectively (Table 3). On the average, 0.92 cm was recorded under GCC for all the 30-long MEL accessions selected, while on the FSC, 1.44 cm was recorded on the soil condition at 5 cm sowing depth for the same set of 30 accessions (Table 3).

**Table 3:** Comparisons of accessions sown under growth chamber condition and field soil condition at 5-cm depth

No	Varieties	GCC	SC-5 cm depth
1	Yuedao46	1.88	1.48
2	yuedao59	1.5	1.80
3	Si4386	1.36	1.52
4	Xiaofenghuang	1.23	1.73
5	Xiangqing	1.19	1.39
6	Fuyu3	1.16	1.47
7	Yuedao20	1.14	1.80
8	Yudao50	1.12	1.58
9	Xiangchuanwuxinbaimi	1.09	1.26
10	Heijing8	1.08	3.1
11	Yuedao70	1.05	1.47
12	Yuedao36	1.03	1.16
13	Yuedao30	0.92	1.79
14	Jiuxiaozhong	0.82	1.09
15	Beidao4	0.81	2.25
16	Kasala	0.79	2.60
17	Yuedao44	0.77	1.16
18	Yuedao5	0.74	0.88
19	Datougui	0.72	1.02
20	Yuedao97	0.72	0.64
21	Yuedao109	0.72	1.63
22	Yuedao48	0.70	0.75
23	Yuedao67	0.67	1.32
24	Yuedao34	0.64	1.56
25	Zhengzhou, Henan	0.63	1.20
26	Nongxiang25	0.62	0.70
27	Xianhui429	0.61	1.29
28	Huangsandannuo	0.61	1.73
29	Yuedao93	0.60	0.97
30	Yuedao31	0.60	0.74
	Average	0.92	1.44

#### Accessions at 2-cm depth (Short MEL)

Thirty accessions selected from the GCC which were classified under short MEL were sowed on the FSC at a depth of 2 cm. Comparing their performance after days of observation, Yuedao22 recorded the highest MEL (0.59 cm) and Yuedao2 had the lowest MEL (0.34 cm) under GCC after 10 days of observation (Table 4). On the FSC, Yuedao7 had the highest MEL (1.78 cm) and Yuedao54 had the lowest MEL (0.22 cm) (Table 4). On the average, 0.43 cm and 0.87 cm were found to be averages for MEL for the 30 accessions classified under short MEL (Table 4). Interestingly, 3 accessions out of 30 accessions (Yuedao7, Yuedao33, Yuedao110) classified under short MEL group have performed above 1.50 cm on the field at 2 cm sowing depth of 1.78 cm, 1.52 cm and 1.51 cm respectively. These results indicate that irrespective of the sowing depth, some accessions have the potential to perform better on the field soil.

**Table 4:** Comparisons of accessions sown under growth chamber condition and field soil condition at 2-cm depth

Varieties	GCC	SC-2 cm depth
Yudao22	0.59	1.12
Shengtangdao	0.56	0.52
Xishihuang	0.56	0.27
Katena	0.56	0.96
Mudanjiang28	0.55	0.6
Yuedao38	0.54	1.15
Yuedao25	0.53	0.31
Yuedao32	0.53	1.21
Yuedao23	0.50	0.44
Yuedao43	0.48	1.47
Yuedao55	0.45	1.16
Yueda3	0.42	0.29
Yuedao56	0.41	0.28
Chuyanghan32	0.40	1.02
Yuedao37	0.40	1.45
Yuedao33	0.39	1.52
Yuedao96	0.39	1.31
Yuedao42	0.39	1.08
Yuedao6	0.37	1.4
Yuedao10	0.37	0.42
Yuedao26	0.37	0.97
Yuedao54	0.36	0.22
Yuedao110	0.36	1.51
Zidao	0.36	0.30
Yuedao94	0.35	1.08
Wuyujing3	0.35	0.32
C418	0.35	0.30
Yuedao7	0.35	1.78
Yuedao51	0.35	1.10
Yuedao2	0.34	0.64
Average	0.43	0.87

### QTLs and favorable alleles detected for MEL in this study

**Fig. 7:**
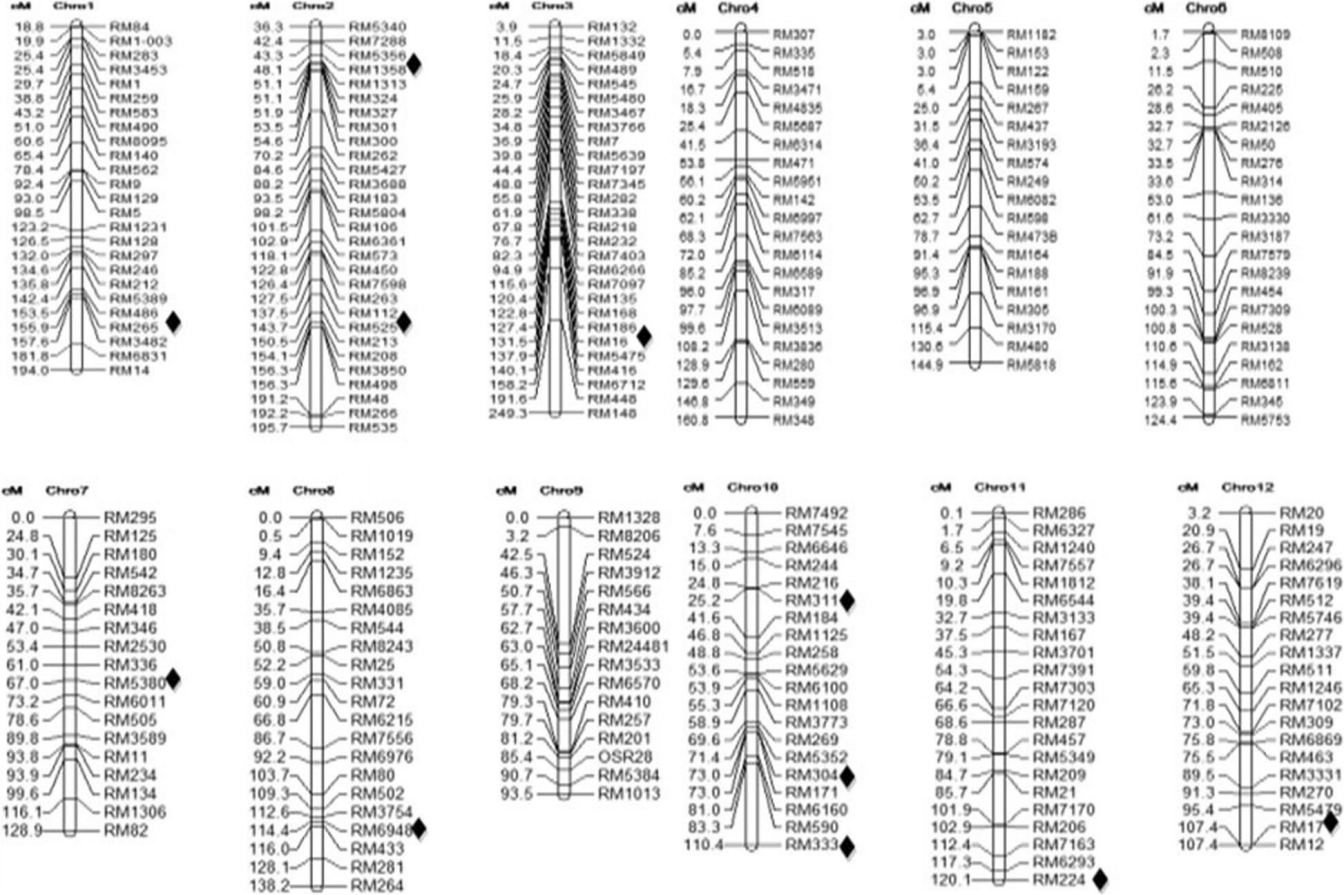
Chromosome position of (measured in cM) QTLs detected for MEL♦

A marker-trait association analysis based on a mixed linear model (MLM) revealed eleven markers/ QTLs were associated with MEL with *P*-value less than 0.01 (Table 5). The MLM analysis revealed 11 marker loci associated with MEL (*P* < 0.01) and the identified markers were located on chromosome 1, 2 (2), 3, 7, 8, 10 (3), 11 and 12 (Table 5). The range of phenotypic variation explained (PVE) was from 4.0% to 11.3%. RM5380 on chromosome 7, which resides on 67 cM, had the maximum PVE for MEL (Table 5). Chromosome position of QTLs detected for MEL in this study as shown in Fig. 7. A summary of all the favorable alleles and their typical carrier materials is shown in Table 6. The total numbers of positive favorable alleles for MEL, detected across the entire population at *P* < 0.05 was 30 (Table 6). The allele RM265-140 bp showed the largest phenotypic effect (1.80 cm) over all the positives alleles for MEL, and the typical carrier accession was Yuedao 46 followed by allele RM16-230 bp with phenotypic effect of 1.3 cm over all the positives alleles for MEL (Table 6). Favorable alleles carried by the superior parent for MEL and corresponding effects were also shown in Table 7.

**Table 5:** Marker-trait associations with *P*-value less than 0.01, QTLs detected, proportion of phenotypic variance explained (PVE), marker position on chromosome derived from 262 markers and 543 rice accessions

Trait	QTL	Chr	Marker linked with QTL	Position (cM)	*P* value	PVE (%)
MEL/cm	qMel-1	1	RM265	170	5.38E-08	9.1
	qMel-2–1	2	RM525	118.1	2.75E-04	10.2
	qMel-2–2	2	RM1358	48.1	2.76E-03	6.1
	qMel-3	3	RM16	84.5	5.20E-05	5.5
	qMel-7	7	RM5380	67	1.21E-06	11.3
	qMel-8	8	RM6948	114.4	1.18E-03	4.6
	qMel-10–1	10	RM304	38.1	1.17E-04	5.7
	qMel-10–2	10	RM311	46.4	6.74E-04	4.8
	qMel-10–3	10	RM333	121.3	2.55E-03	4.0
	qMel-11	11	RM224	117.4	5.92E-03	5.9
	qMel-12	12	RM17	107.4	6.23E-04	9.6

The QTL names are denoted following the rules suggested by McCouch et al. (2008), where the number after the first dash means the chromosome number and the number after the second dash is used to distinguish the two QTL detected on the same chromosome for the same trait

**Table 6:** Positive elite alleles, phenotypic effect value (*P* < 0.05) and typical carrier materials for mesocotyl elongation length

Trait	Locus-allele	Phenotypic effect value	Carrier variety
MEL (cm)	RM 265–140	1.80	Yuedao46
	RM 265–110	0.10	Yuedao57
	RM 265–105	0.10	Xiangqing
	RM 5380–130	1.20	Yuedao59
	RM 5380–115	0.21	Yuedao20
	RM 5380–140	0.19	Yuedao60
	RM 16–230	1.30	Si4386
	RM 304–165	0.17	Si4386
	RM 304–175	0.16	Xiangchuanwuxinbaimi
	RM 304–170	0.16	Nongxiang25
	RM 525–100	0.18	Yuedao59
	RM 525–105	0.22	Fuyu3
	RM 525–145	0.06	Xiangchuanwuxinbaimi
	RM 525–310	1.02	Heijing8
	RM 525–115	0.25	Yuedao70
	RM 525–110	0.08	Huangsandannuo
	RM 525–95	0.19	Yuedao77
	RM 17–185	0.42	Yuedao46
	RM 311–175	0.34	Si4386
	RM 6948–110	0.13	Yuedao46
	RM 6948–235	0.29	Si4386
	RM 6948–220	0.07	Nongxiang25
	RM 1358–175	0.06	Xiaofenghuang
	RM 333–170	0.19	Yuedao20
	RM 333–160	0.23	Yuedao70
	RM 333–190	0.15	Nongxiang25
	RM 224–170	0.41	Yuedao46
	RM 224–135	0.21	Yuedao59
	RM 224–105	0.59	Yuedao109
	RM 224–110	0.29	Yuedao54

**Table 7:** Elite alleles carried by the superior parents for MEL and corresponding phenotypic effect

Trait	Super parent	Locus-allele (Corresponding phenotypic effect value)				
MEL	Yuedao46	RM 265–140(1.8)	RM 304–135(0.01)	RM 17–185(0.42)	RM 6948–110(0.13)	RM 1358–155(0.03)
		RM 224–170(0.41)				
	Yuedao59	RM 5380–130(1.2)	RM 525–100(0.18)	RM 17–160(0.01)	RM 6948–105(0.03)	RM 224–135(0.21)
	Si4386	RM 16–230(1.3)	RM 304–165(0.17)	RM 311–175(0.34)	RM 6948–235(0.29)	
	Xiangchuanwuxinbaimi	RM 525–145(0.06)	RM 304–175(0.16)	RM 5380–110(0.02)		
	Fuyu3	RM 265–115(0.03)	RM 525–105(0.22)	RM 1358–180(0.02)		

### Parental combinations predicted for MEL improvement

Based on the genome distribution of the favorable alleles at eleven significant marker-MEL association loci in typical carrier varieties (Table 7), parental combinations were predicted for improving MEL in rice via cross-breeding based on the data presented (Table 8). For instance, ‘Yuedao46’ had six favourable alleles, and ‘Si4386’ had four favourable alleles, seven favourable alleles could be pyramided into one plant using the combination ‘Yuedao46’ × ‘Si4386’. ‘Yuedao59’ had five favourable alleles and ‘Yuedao46’ will give six favorable alleles which could be pyramided into one plant using the combination ‘Yuedao59’ × ‘Yueado46’ (Table 8).

**Table 8:** Parental combinations, numbers of elite alleles, and phenotypic effects after combinations predicted from association mapping of mesocotyl length and shoot length

Trait	Parental combination	No. elite alleles predicted	MEL improvement predicted (%)
MEL (cm)	Yuedao 59 × Si4386	7	17.43
	Yuedao 59 × Yuedao 46	6	22.70
	Yuedao 46 × Xiangchuanwuxinbaimi	6	25.99
	Yuedao 46 × Si4386	6	16.45
	Yuedao 46 × Yuedao 20	6	17.43

## Discussion

Two new findings were obtained in the present study.

First, it was discovered that among the 543 accessions screened for MEL, only 130 accessions showed mesocotyl elongation length under dark germination condition, indicating that not every accession can elongate its mesocotyl. This means that the QTL and its carrier varieties we detected are precious for our future rice programme development for DDS. Seeding depth is an important factor for dry-direct-seeded rice cultivation and, deep seeding is known to reduce damages from the environment caused by wildlife and improve lodging tolerance. Seeds sown deep need to elongate their organs to ensure that it reaches the soil surface and this was proven in previous experiments conducted by other authors showed that higher MEL served to elevate the coleoptile to allow the primary leaves to emerge and further demonstrate that MEL had a great influence on seedling establishment in rice [39]. The correlation coefficient (*r* = 0.485) between GCC and FSC showed positive relationship and highly significant (*P* < 0.01), indicating that result obtained in GCC could basically represent that obtained under FSC. Although the field soil trial is important in confirming the accessions ability to elongate on the field and actual application in agriculture, the growth chamber trial is a simpler and a direct method for crop breeders to screen resources for MEL.

Second, 7 novel QTLs were detected for mesocotyl elongation length in rice. Among the eleven marker loci associated with MEL, four loci were also reported by previous researchers [5, 10, 13] (Table 9). The 7 new QTLs for MEL were located on chromosomes 2 (2), 7, 8, and 10 (3).

**Table 9:** List for QTLs identified from this study and shared in previous studies

					QTL reported in the previous studies		
Traits	SSR Markers	Chromosomes	Start position/bp	End position/bp	Start position/bp	End position/bp	Reference
MEL/cm	RM265	1	35,196,573	35,196,681	37,713,253	37,713,609	Lee et al. 2012 [5]
	RM16	3	23,126,064	23,126,231	23,088,332	23,088,539	Redona ED, Mackill DJ (1996) [13]
	RM224	11	(117.9–117.9 cM)		(117.9–117.9 cM)		Cai HW, Morishima H (2002) [10]
	RM17	12	26,954,657	26,954,947	23,356,943	23,357,132	Lee et al. 2012 [5]

To improve MEL of rice varieties under dry direct seeding, favourable alleles will have to be pyramided as much as possible into one genotype. Hybridization between varieties carrying favorable alleles and artificial selection should improve MEL in the next step. Predicted according to the results obtained in this study, the combinations ‘Yuedao 59 × Yuedao 46’, ‘Yuedao 46 × Xiangchuanwuxinbaimi’, could theoretically improve the MEL by 0.69 cm and 0.76 cm respectively. (Tables 7 and 8). Four of the five predicted combinations have ‘Yuedao 46’ as a parent, indicating that ‘Yuedao 46’ is a precious resource for MEL improvement.

## Conclusions

Not every genotype of the rice accessions used possesses the ability to elongate its mesocotyl length under dark or deep sowing condition. Mesocotyl elongation length trait is a quantitative trait controlled by many gene loci, and can be improved by pyramiding favorable alleles at different loci into a genotype. The germplasm with favorable alleles for MEL mined here could serve as excellent parents for improving rice cultivars suitable to DDS.

## Supplementary Information

**Additional file 1: Table S1. **Rice materials and their membership probabilities corresponding to each subpopulation.

**Additional file 2: Table S2.** Genotype data(bp) of 543 rice accession amplified by 262 SSR markers.

**Additional file 3: Table S3.** Summary statistics for the 262 SSR markers used in this study.

## Data Availability

All data generated or analysed during this study are included in this published article [Additional file 1: Table S1, Additional file 2: Table S2 and Additional file 3: Table S3].

## Abbreviations

DDS: Dry direct seeding
MLM: Mixed linear model
H^2^_B_: Broad-sense heritability
PIC: Polymorphism information content
PVE: Phenotypic variation explained
LD: Linkage disequilibrium
GCC: Growth chamber condition
