# Supplementary material for: Screening germplasm and detecting QTLs for mesocotyl elongation trait in rice (Oryza sativa L.) by association mapping
Source: BMC Genom Data. 2023 Feb 15;24:8. doi: 10.1186/s12863-023-01107-8 (PMC9930352; doi:10.1186/s12863-023-01107-8)
Supplement: Supplementary file 1 — Additional file 1: Table S1. Rice materials and their membership probabilities corresponding to each subpopulation. [file 12863_2023_1107_MOESM1_ESM.pdf]

Additional file 1: Table S1. Rice materials and their membership probabilities corresponding to each subpopulation

Code	Accessions	Pedigree	Origin	Av MEL	Q values					Sub-pop
					Q1	Q2	Q3	Q4	Q5	
W1	Yazihuang	T045	Jinshan, Shanghai	0.00	0	0.998	0.001	0	0	2
W2	Hongmangzaodao	T261	Kunshan, Jiangsu	0.00	0.024	0.882	0.002	0	0.092	2
W3	Wanhuangdao	T815	Wuxian, Jiangsu	0.00	0	0.999	0.001	0	0	2
W4	Guozinuo	T680	Kunshan, Jiangsu	0.00	0	0.999	0	0	0	2
W5	Shuijingbaidao	T543	Wuxian, Jiangsu	0.00	0	1	0	0	0	2
W6	Wumangzaodao	T442	Changshu, Jiangsu	0.20	0	0.998	0.001	0	0	2
W7	Sanbailitou	T527	Kunshan, Jiangsu	0.00	0	0.999	0	0	0	2
W8	Cuyingwanyangdao	T585	Wuxi, Jiangsu	0.17	0	1	0	0	0	2
W9	Yanglingdao	T632	Wuxi, Jiangsu	0.00	0	1	0	0	0	2
W10	Wanyedao	T643	Wuxian, Jiangsu	0.00	0	1	0	0	0	2
W11	Qiaobinghuang	T684	Taicang, Jiangsu	0.00	0	1	0	0	0	2
W12	Tiejingqing	T651	Kunshan, Jiangsu	0.00	0	1	0	0	0	2
W13	Xiaobaiyedao	T777	Wuxi, Jiangsu	0.00	0	1	0	0	0	2
W14	Baoxintaihuqing	T834	Wujiang, Jiangsu	0.00	0.038	0.932	0.002	0.001	0.027	2
W15	Jiangfeng4	T655	Jiangyin, Jiangsu	0.00	0	0.999	0	0	0	2
W16	Sujing4	T643	Suzhou, Jiangsu	0.00	0	0.999	0	0	0	2
W17	Aizhongluohanhuang	T315	Changshu, Jiangsu	0.00	0	1	0	0	0	2
W18	Baodao	T100	Wuxi, Jiangsu	0.00	0	1	0	0	0	2
W19	Wanmuxiqiu	T101	Taicang, Jiangsu	0.00	0	1	0	0	0	2
W20	Huangsanshi	T528	Wujiang, Jiangsu	0.00	0	1	0	0	0	2
W21	Erheidao	T129	Wuxi, Jiangsu	0.00	0	1	0	0	0	2
W22	Xiaoqingzhong	T737	Wuxian, Jiangsu	0.00	0	1	0	0	0	2
W23	Zaoguangtou	T179	Wuxi, Jiangsu	0.00	0	1	0	0	0	2
W24	Xiaoluohanhuang	T325	Changshu, Jiangsu	0.00	0	1	0	0	0	2
W25	Souzhouqing	T167	Jiangyin, Jiangsu	0.00	0	1	0	0	0	2
W26	Wanluli	T331	Jiangyin, Jiangsu	0.00	0	1	0	0	0	2
W27	Wanbaguo	T357	Jiangyin, Jiangsu	0.00	0	1	0	0	0	2

W28	Ebusinuodao	T386	Wuxi, Jiangsu	0.22	0	1	0	0	0	2
W29	Laodiegu	T397	Wujiang, Jiangsu	0.00	0	1	0	0	0	2
W30	Yefenghuang	T402	Wujiang, Jiangsu	0.00	0	1	0	0	0	2
W31	Chenjiazhong	T554	Kunshan, Jiangsu	0.00	0	1	0	0	0	2
W32	Zaoheitouhong	T473	Wujiang, Jiangsu	0.00	0	1	0	0	0	2
W33	Luohanhuang	T560	Jiangyin, Jiangsu	0.00	0	1	0	0	0	2
W34	Longgouzhong	T580	Qingpu, Shanghai	0.00	0	1	0	0	0	2
W35	Shiluqing	T652	Kunshan, Jiangsu	0.00	0	1	0	0	0	2
W36	Ligengqing	T656	Yixing, Jiangsu	0.00	0	1	0	0	0	2
W37	Heitouhong	T474	Wujiang, Jiangsu	0.00	0	1	0	0	0	2
W38	Laolaihong	T689	Wuxian, Jiangsu	0.00	0	1	0	0	0	2
W39	Erlibie	T701	Wuxian, Jiangsu	0.00	0	1	0	0	0	2
W40	Jinguhuang	T841	Wujiang, Jiangsu	0.00	0	1	0	0	0	2
W41	Cuganhuangdao	T718	Wujiang, Jiangsu	0.00	0	1	0	0	0	2
W42	Zaoshirihuangdao	T728	Wuxian, Jiangsu	0.00	0	1	0	0	0	2
W43	Shengtangqing	T759	Changshu, Jiangsu	0.00	0	1	0	0	0	2
W44	Xiaomandao	T750	Wujiang, Jiangsu	0.00	0	1	0	0	0	2
W45	Shengtangdao	T206	Changshu, Jiangsu	0.56	0	1	0	0	0	2
W46	Wanmandao	T772	Wujiang, Jiangsu	0.00	0	1	0	0	0	2
W47	Nantouzhong	T600	Kunshan, Jiangsu	0.00	0	1	0	0	0	2
W48	Daniaodao	T453	Changshu, Jiangsu	0.00	0	0.999	0	0	0	2
W49	Kongqueqing	T833	Kunshan, Jiangsu	0.31	0.001	0.991	0.005	0	0.003	2
W50	Kaiqing	T232	Kunshan, Jiangsu	0.00	0	0.998	0.001	0	0.001	2
W51	Manyedao	T513	Kunshan, Jiangsu	0.30	0	0.999	0	0	0	2
W52	Baikenuo	T354	Wujiang, Jiangsu	0.00	0	0.921	0	0	0.078	22
W53	Baimangnuo	T033	Wujiang, Jiangsu	0.00	0	0.999	0.001	0	0.001	2
W54	Xiangzhunuo	T452	Changshu, Jiangsu	0.00	0	0.999	0	0	0	2
W55	Yaxienuo	T480	Wuxian, Jiangsu	0.00	0	0.999	0	0	0	2
W56	Xianhui429	T573	Nanjing, Jiangsu	0.61	0	0.999	0	0	0.001	2
W57	Zijianxian3	T504	Nanjing, Jiangsu	0.00	0.001	0.885	0	0	0.114	2
W58	Huangsandannuo	T106	Wuxi, Jiangsu	0.61	0	0.906	0	0	0.093	2

W59	Jia159	Zheshendao2003008	Jiaxing, Zhejiang	0.28	0	0.999	0	0	0	2
W60	Sidao10	ZD-05529	Siyang, Jiangsu	0.00	0	1	0	0	0	2
W61	Wuqiang	ZD-05545	Wujin, Jiangsu	0.00	0	1	0	0	0	2
W62	Wuyujing3	Suzhongshenzidi156	Wujin, Jiangsu	0.35	0	1	0	0	0	2
W63	Xiushui04	GS01009-1990	Nanjing, Jiangsu	0.00	0	1	0	0	0	2
W64	Zhendao88	Suzhongshenzidi 265	Zhenjiang, Jiangsu	0.00	0	0.999	0	0	0	2
W65	Zhendao6	Guoshendao990009	Zhenjiang, Jiangsu	0.00	0	0.999	0	0	0	2
W66	Taijing9	ZD-05548	Taibei,Tianwan	0.00	0	1	0	0	0	2
W67	Taijing16xuanAC	ZD-05549	Taibei,Tianwan	0.00	0	1	0	0	0	2
W68	Taijing16xuanzi	ZD-05550	Taibei,Tianwan	0.00	0	0.999	0	0	0	2
W69	Diantun502xuanzao	09-02598	Kunming, Yunnan	0.26	0.001	0.945	0	0	0.054	2
W70	Hongdao35	ZD-05552	Nanjing, Jiangsu	0.00	0	1	0	0	0	2
W71	Hongdao37	ZD-05553	Nanjing, Jiangsu	0.00	0	1	0	0	0	2
W72	Zijianjingnuo	ZD-05556	Nanjing, Jiangsu	0.00	0	1	0	0	0	2
W73	Nannongjing62401	ZD-05557	Nanjing, Jiangsu	0.26	0	0.931	0	0.001	0.067	2
W74	Tongjing109	SS201115	Nantong, Jiangsu	0.00	0	0.999	0	0	0	2
W75	Yangdao6	Guoshendao2001002	Yangzhou, Jiangsu	0.37	0.001	0.966	0	0	0.033	2
W76	Nignjing1	Sushendao200417	Nanjing, Jiangsu	0.00	0	1	0	0	0	2
W77	Wujing15	Sushendao200418	Wujin, Jiangsu	0.00	0	1	0	0	0	2
W78	Wuxiangjing14	Sushendao200315	Wujin, Jiangsu	0.00	0	1	0	0	0	2
W79	Xudao3	Sushendao200306	Xuzhou, Jiangsu	0.00	0	1	0	0	0	2
W80	Nannongjing003	ZD-05572	Nanjing, Jiangsu	0.00	0	1	0	0	0	2
W81	Nannongjing005	ZD-05574	Nanjing, Jiangsu	0.00	0	1	0	0	0	2
W82	5jing20	T718	Nanjing, Jiangsu	0.00	0	0.999	0	0	0	2
W83	5jing15	ZD-05575	Nanjing, Jiangsu	0.00	0	1	0	0	0	2
W84	molingjing	ZD-05577	Nanjing, Jiangsu	0.00	0	1	0	0	0	2
W85	5jing03	ZD-05578	Nanjing, Jiangsu	0.00	0	1	0	0	0	2
W86	5jing68	ZD-05579	Nanjing, Jiangsu	0.00	0	1	0	0	0	2
W87	Xudao4	ZD-05646	Xuzhou, Jiangsu	0.00	0	1	0	0	0	2
W88	Xudao5	Guoshendao2006059	Xuzhou, Jiangsu	0.00	0	1	0	0	0	2
W89	Huaidao9	SS200607	Huaian, Jiangsu	0.26	0	1	0	0	0	2

W90	Yandao6	Sushendao200205	Yancheng, Jiangsu	0.21	0	1	0	0	0	2
W91	Yangguang200	Guoshendao2008043	Lianyungang, Jiangsu	0.00	0	1	0	0	0	2
W92	Lianjing2	Guoshendao990021	Lianyungang, Jiangsu	0.00	0	1	0	0	0	2
W93	Xiushui79	Guoshendao2008021	Jiaxing, Zhejiang	0.17	0	0.999	0	0	0	2
W94	Cbao	W92010108	Hefei, Anhui	0.00	0	0.999	0	0	0	2
W95	Nipponbare	Yamabiko/Saikaze	Aichi, Japan	0.00	0	0.899	0.101	0	0	2
W96	Zhen9424	ZD-05658	Zhenjiang, Jiangsu	0.00	0	0.738	0	0.262	0	2
W97	Wuyujing7	SZS300	Wujin, Jiangsu	0.00	0	0	0.701	0.299	0	3
W98	Yanfujing8	Sushendao200608	Yancheng, Jiangsu	0.18	0	0	0.999	0	0	3
W99	Zhengdao18	Sushendao201311	Zhenzhou, Henan	0.26	0	0.001	0.999	0	0	3
W100	Huaidao18	Sushendao201505	Huaian, Jiangsu	0.24	0	0	0.999	0	0	3
W101	Shengdao16	Guoshendao2010048	Jiaxiang, Shandong	0.00	0	0	1	0	0	3
W102	Shengdao14	Lunongshen2007024	Jiaxiang, Shandong	0.00	0	0	0.999	0	0	3
W103	Yujing6	Guoshendao980002	Zhenzhou, Henan	0.00	0	0	1	0	0	3
W104	Huaidao8	Sushendao200410	Huaian, Jiangsu	0.00	0	0	0.999	0	0	3
W105	Jindao9618	Guoshendao2008044	Dongli, Tianjin	0.16	0	0	1	0	0	3
W106	Wandao68	WPS03010384	Hefei, Anhui	0.26	0	0	0.999	0	0	3
W107	Xudao2	SS201411	Xuzhou, Jiangsu	0.00	0	0	0.999	0	0	3
W108	Sujing8	Sushendao200612	Suzhou, Jiangsu	0.00	0	0	0.999	0	0	3
W109	Xiangjing9407	Xiangjing1/82-1244	Nanjing, Jiangsu	0.00	0	0	1	0	0	3
W110	Zhongjing212	unknown	Nanjing, Jiangsu	0.00	0	0	0.999	0	0	3
W111	Zhongjing9677	unknown	Nanjing, Jiangsu	0.00	0	0	0.999	0	0	3
W112	Zhongjing131	unknown	Nanjing, Jiangsu	0.00	0	0	0.999	0	0	3
W113	Suwujing	unknown	Wujin, Jiangsu	0.00	0	0	1	0	0	3
W114	Zhognjing438	unknown	Nanjing, Jiangsu	0.00	0	0	0.999	0	0	3
W115	Yanjing9	SS200707	Yancheng, Jiangsu	0.00	0	0	1	0	0	3
W116	Yangfujing7	Sushendao200413	Yangzhou, Jiangsu	0.00	0	0	0.999	0	0	3
W117	Zijing	09-02587	Nanjing, Jiangsu	0.16	0	0	0.999	0	0	3
W118	Zhendao10	SS200710	Zhenjiang, Jiangsu	0.37	0	0.001	0.999	0	0	3
W119	Zhenghan2	Guoshendao2003031	Zhengzhou, Henan	0.00	0	0	0.999	0	0	3
W120	Zhenghan6	Guoshendao2005055	Zhengzhou, Henan	0.63	0	0	0.999	0	0	3

W121	Xishihuang	T204	Wuxian, Jiangsu	0.56	0	0	1	0	0	3
W122	Daliangdao	T227	Wuxi, Jiangsu	0.00	0	0	1	0	0	3
W123	Heizuidao	T262	Changshu, Jiangsu	0.00	0	0	1	0	0	3
W124	Xiaohuangdao	T288	Wuxian, Jiangsu	0.00	0	0	0.999	0	0	3
W125	Fenghaungdao	T526	Changshu, Jiangsu	0.00	0	0	0.999	0	0	3
W126	Maijieqing	T75	Songjiang, Shanghai	0.00	0	0	1	0	0	3
W127	Jijingdao	T235	Wujiang, Jiangsu	0.00	0	0	1	0	0	3
W128	Zhognsuyangzhogndao	T343	Wuxi, Jiangsu	0.00	0	0	1	0	0	3
W129	Duiguzhong	T368	Wujiang, Jiangsu	0.00	0	0	1	0	0	3
W130	Shuaishaban	T629	Songjiang, Shanghai	0.00	0	0	1	0	0	3
W131	Niumaohuang	T167	Taicang, Jiangsu	0.00	0	0	0.999	0	0	3
W132	Wanheitouhong	T132	Wujiang, Jiangsu	0.00	0	0	1	0	0	3
W133	Taihuqing	T2	Kunshan, Jiangsu	0.00	0	0	0.999	0	0	3
W134	Yilimang	T583	Changshu, Jiangsu	0.00	0	0	0.999	0	0	3
W135	Wuqitou	T470	Wujin, Jiangsu	0.00	0	0	1	0	0	3
W136	Jiaoaiheitouhong	T136	Wujiang, Jiangsu	0.00	0	0	1	0	0	3
W137	Laowusi	T607	Wujiang, Jiangsu	0.00	0	0	1	0	0	3
W138	Manliuzhong	T386	Jinshan, Shanghai	0.00	0	0	1	0	0	3
W139	Tainluohuang	T200	Changshu, Jiangsu	0.00	0	0	1	0	0	3
W140	Jiucaiqing	T73	Changshu, Jiangsu	0.00	0	0	1	0	0	3
W141	Aiqidaliuzhong	T375	Jiading, Shanghai	0.00	0	0	1	0	0	3
W142	Lujingqing	T45	Wujiang, Jiangsu	0.25	0	0	1	0	0	3
W143	Gaoliangqing	T67	Wujiang, Jiangsu	0.00	0	0	1	0	0	3
W144	Yishixing	T562	Changshu, Jiangsu	0.25	0	0	1	0	0	3
W145	Heizhong	T845	Wuxian, Jiangsu	0.00	0	0	1	0	0	3
W146	Louhanbai	T545	Kunshan, Jiangsu	0.26	0	0	0.999	0	0	3
W147	Xueliqing	T31	Wuxi, Jiangsu	0.00	0	0	1	0	0	3
W148	Liyangxiaohongdao	T94	Liyang, Jiangsu	0.00	0	0	0.999	0	0	3
W149	Juhuahuang	T185	Wuxi, Jiangsu	0.00	0	0	1	0	0	3
W150	Changzijingyedao	T332	Wuxian, Jiangsu	0.00	0	0	1	0	0	3
W151	Gaidaoqing	T61	Wujiang, Jiangsu	0.00	0	0	0.864	0	0.135	3

W152	Dingzhuangdao	T252	Wuxi, Jiangsu	0.00	0	0	1	0	0	3
W153	Xuetangzhong	T351	Jiangyin, Jiangsu	0.00	0	0	1	0	0	3
W154	Guanbaidan	T580	Wujiang, Jiangsu	0.00	0	0	1	0	0	3
W155	Hongbaodao	T145	Jiaxing, Zhejiang	0.00	0	0	1	0	0	3
W156	Tiegandao	T272	Wujiang, Jiangsu	0.00	0	0	0.999	0	0	3
W157	Juzigaung	T608	Wuxi, Jiangsu	0.00	0	0	0.925	0	0.075	3
W158	Yebaidao	T321	Taicang, Jiangsu	0.00	0	0	1	0	0	3
W159	Daheitouhong	T137	Wujiang, Jiangsu	0.00	0	0	1	0	0	3
W160	Baigedao	T551	Wuxian, Jiangsu	0.00	0	0	1	0	0	3
W161	Diediezhong	T356	Qingpu, Shanghai	0.00	0	0	1	0	0	3
W162	Puxidadaotou	T482	Wujiang, Jiangsu	0.00	0	0	1	0	0	3
W163	Yangdao	T339	Wujiang, Jiangsu	0.00	0	0	1	0	0	3
W164	Yanhongdao	T144	Wujiang, Jiangsu	0.00	0	0	0.556	0.444	0	3
W165	Baikewandao	T275	Wuxi, Jiangsu	0.00	0	0	1	0	0	3
W166	Aiguodadaotou	T460	Wujiang, Jiangsu	0.00	0	0	1	0	0	3
W167	Sishitou	T577	Wuxian, Jiangsu	0.00	0	0	1	0	0	3
W168	Jiuxiaozhong	T425	Wujiang, Jiangsu	0.82	0	0	1	0	0	3
W169	Chushuhuang	T182	Wuxian, Jiangsu	0.00	0	0	1	0	0	3
W170	Qianjindao	T116	Wujiang, Jiangsu	0.33	0	0	1	0	0	3
W171	Qijiangqing	T50	Kunshan, Jiangsu	0.31	0	0	1	0	0	3
W172	Baishuqing	T72	Qingpu, Shanghai	0.00	0	0	1	0	0	3
W173	Feilaifeng	T519	Wuxi, Jiangsu	0.00	0	0	1	0	0	3
W174	Kejia6	Y11	Kunshan, Jiangsu	0.00	0	0	1	0	0	3
W175	Lamujia	Y14	Kunshan, Jiangsu	0.00	0	0	1	0	0	3
W176	Haonuopie	Y42	Kunshan, Jiangsu	0.00	0	0	1	0	0	3
W177	Xiganggu	Y47	Wujiang, Jiangsu	0.00	0	0	1	0	0	3
W178	Shuangchengnuo	Y71	Wujiang, Jiangsu	0.00	0	0	1	0	0	3
W179	Qiutiandaxiedao	Y73	Wujiang, Jiangsu	0.00	0	0	1	0	0	3
W180	Qiyunuo10	Y74	Wujiang, Jiangsu	0.00	0	0	1	0	0	3
W181	Wunuo1	Y82	Wujiang, Jiangsu	0.00	0	0	1	0	0	3
W182	Jianongnuo2	Y103	Wujiang, Jiangsu	0.29	0	0	1	0	0	3

W183	Hongnong5	Y129	Wujiang, Jiangsu	0.00	0	0	0.999	0	0	3
W184	Nonglinnuo4	Y142	Wujiang, Jiangsu	0.00	0	0	0.999	0	0	3
W185	Xiangnuodao	Y143	Wuxian, Jiangsu	0.00	0	0	0.999	0	0	3
W186	Luchaihong	Y149	Wujiang, Jiangsu	0.00	0	0	0.999	0	0.001	3
W187	Cungu	Y164	Wujiang, Jiangsu	0.32	0	0	0.968	0	0.031	3
W188	Katena	Y165	Wujiang, Jiangsu	0.56	0	0	0.999	0	0.001	3
W189	Guanchanuo	Y167	Wujiang, Jiangsu	0.00	0	0	0.997	0	0.001	3
W190	Kuihuanuo	Y185	Wuxian, Jiangsu	0.00	0	0	0.935	0	0.065	3
W191	Suyunuo	Y226	Wuxian, Jiangsu	0.00	0	0	0.999	0	0	3
W192	Hongjiaozhan	Y297	Wuxian, Jiangsu	0.00	0.001	0	0.888	0	0.11	3
W193	Haobuka	Y317	Wuxian, Jiangsu	0.00	0	0.001	0.842	0	0.156	3
W194	Chuyanghan32	Y341	Wuxian, Jiangsu	0.40	0.001	0.002	0.851	0	0.146	3
W195	Libanyi	Y343	Wuxian, Jiangsu	0.20	0.001	0	0.885	0	0.114	3
W196	Kunnong8	T649	Kunshan, Jiangsu	0.00	0	0	0.997	0.001	0.001	3
W197	Guihuahuang	T641	Nanjing, Jiangsu	0.00	0	0	0.999	0	0	3
W198	Zhoujiazhong	T687	Wujiang, Jiangsu	0.15	0	0	1	0	0	3
W199	Xiaofenghuang	T529	Wuxian, Jiangsu	1.23	0	0	1	0	0	3
W200	Xiangjingdao	T724	Wuxian, Jiangsu	0.00	0	0	1	0	0	3
W201	Huizao	T591	Wujiang, Jiangsu	0.00	0	0	0.999	0	0	3
W202	Yingtoudao	T270	Kunshan, Jiangsu	0.00	0	0	1	0	0	3
W203	Changdaotou	T465	Wujiang, Jiangsu	0.00	0	0	1	0	0	3
W204	Yangmiaozhong	T440	Wujiang, Jiangsu	0.00	0	0	1	0	0	3
W205	Maoguangdao	T247	Wuxian, Jiangsu	0.00	0	0	1	0	0	3
W206	Dazhongdao	T266	Wujiang, Jiangsu	0.00	0	0	1	0	0	3
W207	Sanxiadao	T232	Wuxi, Jiangsu	0.00	0	0	1	0	0	3
W208	Xiaoqingmang	T604	Changshu, Jiangsu	0.00	0	0	1	0	0	3
W209	Hongganlizhihong	T123	Wujiang, Jiangsu	0.00	0	0	1	0	0	3
W210	Wuxidao	T256	Changshu, Jiangsu	0.00	0	0	1	0	0	3
W211	Wanzhogngiu	T601	Wuxian, Jiangsu	0.00	0	0	1	0	0	3
W212	Fengjingdao	T273	Wuxian, Jiangsu	0.00	0	0	1	0	0	3
W213	Liuzhong	T369	Changshu, Jiangsu	0.00	0	0	1	0	0	3

W214	Cuganlizhihong	T122	Wuxian, Jiangsu	0.00	0	0	1	0	0	3
W215	Chiguwandao	T147	Wujiang, Jiangsu	0.29	0	0	1	0	0	3
W216	Jiaobaiyeqing	T76	Songjiang, Shanghai	0.00	0	0	0.879	0.001	0.12	3
W217	Chiguhong	T148	Wujiang, Jiangsu	0.00	0	0	0.998	0	0.001	3
W218	Fanluoqing	T51	Kunshan, Jiangsu	0.00	0	0	1	0	0	3
W219	Zaoyedao	T322	Kunshan, Jiangsu	0.00	0	0	1	0	0	3
W220	Baidiegu	T361	Wujiang, Jiangsu	0.00	0	0	1	0	0	3
W221	Wangjiadao	T258	Wujiang, Jiangsu	0.00	0	0	1	0	0	3
W222	Jiangyinzhong	T447	Jiangyin, Jiangsu	0.00	0	0	1	0	0	3
W223	Eyingbaijingdao	T279	Jiading, Shanghai	0.00	0	0	1	0	0	3
W224	Tiekewanguangtou	T487	Wujin, Jiangsu	0.00	0	0	1	0	0	3
W225	Tiekedao	T271	Wujin, Jiangsu	0.00	0	0	1	0	0	3
W226	Dadaosuitou	T462	Changshu, Jiangsu	0.00	0	0	1	0	0	3
W227	Aibaidao	T265	Wujiang, Jiangsu	0.00	0	0	1	0	0	3
W228	Xiepihuang	T665	Taicang, Jiangsu	0.00	0	0	1	0	0	3
W229	Xiaobaidao	T667	Wuxian, Jiangsu	0.00	0	0	1	0	0	3
W230	Baishidao	T222	Taicang, Jiangsu	0.00	0	0	1	0	0	3
W231	Manbaidao	T215	Wujiang, Jiangsu	0.00	0	0	1	0	0	3
W232	Guangtouluhuabai	T540	Wuxi, Jiangsu	0.00	0	0	1	0	0	3
W233	Hongmangjing	T630	Kunshan, Jiangsu	0.00	0	0	1	0	0	3
W234	Wumangyedao	T335	Changshu, Jiangsu	0.21	0	0	0.999	0	0	3
W235	Luhuabai	T531	Wuxian, Jiangsu	0.00	0	0	0.999	0	0	3
W236	Haidongqing	T19	Kunshan, Jiangsu	0.00	0	0	1	0	0	3
W237	Shenlenuo	Y26	Kunshan, Jiangsu	0.00	0	0	0.999	0	0	3
W238	Xiangqing	XiangT302(♀) / Qingsan2377(♂)	Chongming, Shanghai	1.19	0	0	0.962	0	0.037	3
W239	Jinghui418	Wanlun422/Miyang23	Shenyang, Liaoning	0.00	0.001	0	0.922	0	0.077	3
W240	Malaihong	unknown	Nanjing, Jiangsu	0.00	0	0	0.86	0	0.139	3
W241	Jingnuo4921	Wanpingshen99010256	Hefei, Anhui	0.00	0	0	0.259	0.728	0.012	4
W242	Huadao6	Suzhongshenzi354	Huaian, Jiangsu	0.00	0	0	0.274	0.725	0	4
W243	Fuyu3	unknown	Yuexi, Anhui	1.16	0	0	0.109	0.743	0.148	4
W244	Dongnongjing424	Heishendao2005002	Shenyang, Liaoning	0.00	0	0	0.001	0.997	0.002	4

W245	Dongnong9006	Heishendao2013016	Shenyang, Liaoning	0.00	0	0	0.131	0.868	0	4
W246	R254	Xiangqing / Kouxianghong	Chongming, Shanghai	0.00	0	0	0.202	0.797	0	4
W247	Jiangyinnuo	T867	Jiangyin, Jiangsu	0.00	0	0	0.198	0.801	0	4
W248	Jinggunuo	T853	Wuxi, Jiangsu	0.00	0	0	0.214	0.785	0	4
W249	Shanhonggu	T103	Wujiang, Jiangsu	0.00	0	0	0.198	0.802	0	4
W250	Wanshengmaohuang	T169	Wuxi, Jiangsu	0.00	0	0	0.225	0.774	0	4
W251	Wanyangdao	T345	Wuxian, Jiangsu	0.33	0	0	0.216	0.784	0	4
W252	Aidazhong	T407	Wujiang, Jiangsu	0.33	0	0	0.227	0.773	0	4
W253	Jijiaohong	T141	Wuxian, Jiangsu	0.00	0	0	0.201	0.798	0	4
W254	Toulaizhong	T426	Wujiang, Jiangsu	0.00	0	0	0.001	0.999	0	4
W255	Huakenuo	T815	Wujiang, Jiangsu	0.00	0	0	0	1	0	4
W256	Toudengyishixing	T714	Kunshan, Jiangsu	0.00	0	0	0	1	0	4
W257	Maozitou	T897	Wujiang, Jiangsu	0.00	0	0	0	1	0	4
W258	Zaonuodao	T868	Wujiang, Jiangsu	0.27	0	0	0	1	0	4
W259	Datougui	T902	Changshu, Jiangsu	0.72	0	0	0	1	0	4
W260	Zaoxiaobaidao	T709	Wuxi, Jiangsu	0.00	0	0	0	1	0	4
W261	Kangzhounuo	T769	Wujiang, Jiangsu	0.00	0	0	0	1	0	4
W262	Kuobanzhong	T383	Qingpu, Shanghai	0.00	0	0	0	1	0	4
W263	Yangzhongdao	T342	Wujiang, Jiangsu	0.00	0	0	0	1	0	4
W264	Huangkewanguangtou	T486	Wujin, Jiangsu	0.00	0	0	0	1	0	4
W265	Tiehanyishixing	T569	Wuxi, Jiangsu	0.00	0	0	0	1	0	4
W266	Aijiaoluganhuang	T190	Changshu, Jiangsu	0.00	0	0	0	1	0	4
W267	Zhonghua3	Y235	Haidian, Beijing	0.00	0	0	0	1	0	4
W268	Buxienuo	Y256	kunshan, Jiangsu	0.00	0	0	0	1	0	4
W269	Wandao68	Wanpinshen03010384	Hefei, Anhui	0.00	0	0	0	1	0	4
W270	C418	GS2004046	Shenyang, Liaoning	0.35	0	0	0	1	0	4
W271	Fuxiang1	GS2009029	Yuexi, Anhui	0.00	0	0	0	1	0	4
W272	Yuedao1	YD001	Vietnam	0.00	0	0	0	0.806	0.193	4
W273	Yuedao2	YD002	Vietnam	0.34	0	0	0	0.688	0.312	4
W274	Yuedao3	YD003	Vietnam	0.42	0	0	0	0.672	0.327	4
W275	Yuedao4	YD004	Vietnam	0.00	0	0	0	0.67	0.33	4

W276	Yuedao5	YD005	Vietnam	0.74	0	0	0	0.65	0.35	4
W277	Yuedao6	YD006	Vietnam	0.00	0	0	0	0.654	0.346	4
W278	Yuedao7	YD007	Vietnam	0.35	0	0	0	0.628	0.371	4
W279	Yuedao8	YD008	Vietnam	0.00	0	0	0	0.647	0.352	4
W280	Yuedao9	YD009	Vietnam	0.25	0	0	0	0.638	0.362	4
W281	Yuedao10	YD010	Vietnam	0.25	0	0	0	0.638	0.362	4
W282	Yuedao11	YD011	Vietnam	0.00	0	0	0	0.653	0.346	4
W283	Yuedao12	YD012	Vietnam	0.00	0	0	0	0.644	0.355	4
W284	Yuedao13	YD013	Vietnam	0.00	0	0	0	0.654	0.346	4
W285	Yuedao14	YD014	Vietnam	0.00	0	0	0	0.629	0.37	4
W286	Yuedao15	YD015	Vietnam	0.00	0	0	0	0.648	0.352	4
W287	Yuedao16	YD016	Vietnam	0.00	0	0	0	0.65	0.35	4
W288	Yuedao17	YD017	Vietnam	0.00	0	0	0	0.655	0.345	4
W289	Yuedao18	YD018	Vietnam	0.00	0	0	0	0.029	0.97	5
W290	Yuedao19	Y1A02320	Vietnam	0.00	0	0	0	0	1	5
W291	Yuedao20	Y1A02387	Vietnam	1.14	0	0	0	0	1	5
W292	Yuedao21	YD021	Vietnam	0.25	0	0	0	0	1	5
W293	Yuedao22	YD022	Vietnam	0.59	0	0	0	0	1	5
W294	Yuedao23	Y1A02321	Vietnam	0.50	0	0	0	0	0.999	5
W295	Yuedao24	Y1A02322	Vietnam	0.31	0	0	0	0	1	5
W296	Yuedao25	YD025	Vietnam	0.53	0	0	0	0	1	5
W297	Yuedao26	YD026	Vietnam	0.37	0	0	0	0	1	5
W298	Yuedao27	Y1A02323	Vietnam	0.00	0	0	0	0	1	5
W299	Yuedao28	Y1A02324	Vietnam	0.27	0	0	0	0	1	5
W300	Yuedao29	YD029	Vietnam	0.00	0	0	0	0	1	5
W301	Yuedao30	YD030	Vietnam	0.92	0	0	0	0	1	5
W302	Yuedao31	Y1A02325	Vietnam	0.60	0	0	0	0	1	5
W303	Yuedao32	Y1A02326	Vietnam	0.53	0	0	0	0	1	5
W304	Yuedao33	YD033	Vietnam	0.39	0	0	0	0	1	5
W305	Yuedao34	Y1A02327	Vietnam	0.64	0	0	0	0	1	5
W306	Yuedao35	YD035	Vietnam	0.00	0	0	0	0	1	5

W307	Yuedao36	YD36	Vietnam	1.03	0	0	0	0	1	5
W308	Yuedao37	YD37	Vietnam	0.40	0	0	0	0	1	5
W309	Yuedao38	YD38	Vietnam	0.54	0	0	0	0	1	5
W310	Yuedao39	YD39	Vietnam	0.17	0	0	0	0	1	5
W311	Yuedao40	YD40	Vietnam	0.15	0	0	0	0	1	5
W312	Yuedao41	Y1A02328	Vietnam	0.00	0	0	0	0	1	5
W313	Yuedao42	YD42	Vietnam	0.39	0	0	0	0	1	5
W314	Yuedao43	YD43	Vietnam	0.48	0	0	0	0	1	5
W315	Yuedao44	YD44	Vietnam	0.77	0	0	0	0	1	5
W316	Yuedao45	Y1A02329	Vietnam	0.28	0	0	0	0	1	5
W317	Yuedao46	Y1A02330	Vietnam	1.88	0	0	0	0	1	5
W318	Yuedao47	YD47	Vietnam	0.33	0	0	0	0	1	5
W319	Yuedao48	YD48	Vietnam	0.70	0	0	0	0	1	5
W320	Yuedao49	YD49	Vietnam	0.00	0	0	0	0	1	5
W321	Yuedao50	Y1A02331	Vietnam	1.12	0	0	0	0	1	5
W322	Yuedao51	Y1A02332	Vietnam	0.35	0	0	0	0	1	5
W323	Yuedao52	YD52	Vietnam	0.25	0	0	0	0	1	5
W324	Yuedao53	YD53	Vietnam	0.23	0	0	0	0	1	5
W325	Yuedao54	Y1A02335	Vietnam	0.36	0	0	0	0.296	0.703	5
W326	Yuedao55	YD55	Vietnam	0.45	0	0	0	0.09	0.91	5
W327	Yuedao56	YD56	Vietnam	0.41	0	0	0	0	0.999	5
W328	Yuedao57	Y1A02336	Vietnam	0.00	0	0	0	0	1	5
W329	Yuedao58	Y1A02337	Vietnam	0.00	0	0	0	0	1	5
W330	Yuedao59	YD59	Vietnam	1.50	0	0	0	0	1	5
W331	Yuedao60	YD60	Vietnam	0.25	0	0	0	0	1	5
W332	Yuedao61	YD61	Vietnam	0.26	0	0	0	0	1	5
W333	Yuedao62	YD62	Vietnam	0.00	0	0	0	0	1	5
W334	Yuedao63	YD63	Vietnam	0.00	0	0	0	0	0.999	5
W335	Yuedao64	Y1A02338	Vietnam	0.15	0	0.002	0.005	0.004	0.988	5
W336	Yuedao65	YD65	Vietnam	0.34	0	0	0	0	0.999	5
W337	Yuedao66	YD66	Vietnam	0.00	0	0	0.001	0	0.998	5

W338	Yuedao67	Y1A02339	Vietnam	0.67	0	0	0	0	0.999	5
W339	Yuedao68	YD68	Vietnam	0.00	0	0	0	0	1	5
W340	Yuedao69	YD69	Vietnam	0.00	0	0	0	0	1	5
W341	Yuedao70	YD70	Vietnam	1.05	0	0	0	0	1	5
W342	Yuedao71	Y1A02340	Vietnam	0.00	0	0	0	0	1	5
W343	Yuedao72	Y1A02341	Vietnam	0.32	0	0	0	0	1	5
W344	Yuedao73	YD73	Vietnam	0.00	0	0	0	0	1	5
W345	Yuedao74	YD74	Vietnam	0.00	0	0	0	0	1	5
W346	Yuedao75	Y1A02342	Vietnam	0.00	0	0	0	0	1	5
W347	Yuedao76	YD76	Vietnam	0.00	0	0	0	0	1	5
W348	Yuedao77	Y1A02389	Vietnam	0.30	0	0	0	0	1	5
W349	Yuedao78	Y1A02343	Vietnam	0.00	0	0.001	0.001	0	0.998	5
W350	Yuedao79	YD79	Vietnam	0.00	0	0	0	0	0.999	5
W351	Yuedao80	Y1A02390	Vietnam	0.00	0	0	0	0	1	5
W352	Yuedao81	Y1A02344	Vietnam	0.00	0	0	0	0	1	5
W353	Yuedao82	YD82	Vietnam	0.00	0	0	0	0	1	5
W354	Yuedao83	YD83	Vietnam	0.00	0	0	0	0	1	5
W355	Yuedao84	Y1A02391	Vietnam	0.00	0	0	0	0.292	0.707	5
W356	Yuedao85	Y1A02345	Vietnam	0.00	0	0	0	0.061	0.939	5
W357	Yuedao86	YD86	Vietnam	0.23	0	0.001	0.001	0	0.997	5
W358	Yuedao87	YD87	Vietnam	0.18	0	0	0	0	0.999	5
W359	Yuedao88	Y1A02346	Vietnam	0.00	0	0	0	0	1	5
W360	Yuedao89	Y1A02347	Vietnam	0.27	0	0	0	0	1	5
W361	Yuedao90	Y1A02392	Vietnam	0.00	0	0	0	0	1	5
W362	Yuedao91	Y1A02393	Vietnam	0.00	0	0	0	0	1	5
W363	Yuedao92	YD92	Vietnam	0.00	0	0	0	0	1	5
W364	Yuedao93	Y1A02394	Vietnam	0.60	0	0	0	0	1	5
W365	Yuedao94	YD94	Vietnam	0.35	0	0	0	0	1	5
W366	Yuedao95	YD95	Vietnam	0.00	0	0	0	0	1	5
W367	Yuedao96	YD96	Vietnam	0.39	0	0	0.004	0	0.996	5
W368	Yuedao97	YD97	Vietnam	0.72	0	0	0	0	1	5

W369	Yuedao98	Y1A02348	Vietnam	0.00	0	0	0	0	1	5
W370	Yuedao99	Y1A02349	Vietnam	0.27	0	0	0	0	1	5
W371	Yuedao100	Y1A02350	Vietnam	0.00	0	0	0	0	1	5
W372	Yuedao101	Y1A02351	Vietnam	0.00	0	0	0	0	1	5
W373	Yuedao102	Y1A02352	Vietnam	0.00	0	0	0	0	1	5
W374	Yuedao103	YD103	Vietnam	0.00	0	0	0	0	1	5
W375	Yuedao104	Y1A02353	Vietnam	0.00	0	0	0	0	1	5
W376	Yuedao105	Y1A02396	Vietnam	0.00	0	0	0	0	1	5
W377	Yuedao106	Y1A02354	Vietnam	0.00	0	0	0	0	1	5
W378	Yuedao107	YD107	Vietnam	0.34	0	0	0	0	1	5
W379	Yuedao108	Y1A02355	Vietnam	0.24	0	0	0.006	0	0.993	5
W380	Yuedao109	Y1A02356	Vietnam	0.72	0	0.012	0.033	0.003	0.952	5
W381	Yuedao110	YD110	Vietnam	0.36	0	0	0	0	0.999	5
W382	Yuedao111	Y1A02398	Vietnam	0.00	0	0	0	0	1	5
W383	Yuedao112	Y1A02357	Vietnam	0.00	0	0	0	0	1	5
W384	Yuedao113	YD113	Vietnam	0.00	0	0	0	0	1	5
W385	Yuedao114	Y1A02399	Vietnam	0.00	0	0	0.001	0.486	0.513	5
W386	Yuedao115	Y1A02358	Vietnam	0.17	0	0	0.001	0.555	0.443	4
W387	Yuedao116	YD116	Vietnam	0.16	0	0	0.001	0.555	0.444	4
W388	Yuedao117	YD117	Vietnam	0.00	0	0	0	0.564	0.435	4
W389	Yuedao118	YD118	Vietnam	0.00	0	0	0	0.589	0.411	4
W390	Yuedao119	Y1A02359	Vietnam	0.00	0	0	0.004	0.693	0.302	4
W391	Yuedao120	Y1A02360	Vietnam	0.00	0	0.001	0.001	0.625	0.373	4
W392	Yuedao121	Y1A02361	Vietnam	0.00	0	0	0.001	0.63	0.369	4
W393	Jia45	Jia25/D293//Jia25	Jiaxing, Zhejiang	0.00	0	0	0	0.929	0.07	4
W394	Nannongjing3786	Guoshendao2008029	Nanjing, Jiangsu	0.23	0	0	0	0.999	0	4
W395	2428	ZD-05542	Nanjing, Jiangsu	0.00	0	0	0	0.999	0	4
W396	Nannongjing4004	Xihai26/fengqianben	Nanjing, Jiangsu	0.00	0	0	0	0.999	0	4
W397	Nannongjing4016	Akei103B/(Aoisora/Kitariku103)F1	Nanjing, Jiangsu	0.00	0	0	0	0.999	0	4
W398	Zijianwujing	Wuyujing3/Zidao	Nanjing, Jiangsu	0.00	0	0	0	0.999	0	4
W399	Ningjing2	WPS05010476	Nanjing, Jiangsu	0.00	0	0	0	0.999	0	4

W400	Wuxiang99-8	M-02	Wujin, Jiangsu	0.00	0	0	0	0.999	0	4
W401	Wuyujing8	SZS313	Wujin, Jiangsu	0.00	0	0	0	0.999	0	4
W402	Nannongjing002	unknown	Nanjing, Jiangsu	0.00	0	0	0	0.999	0	4
W403	Nannongjing004	unknown	Nanjing, Jiangsu	0.00	0	0	0	1	0	4
W404	Huaidao5hao	SZS358	Huaian, Jiangsu	0.00	0	0	0	1	0	4
W405	Zhongzuo93	Jinshendao1995001	Tongzhou, Beijing	0.00	0	0	0	1	0	4
W406	Yandao9	SS200506	Yancheng, Jiangsu	0.00	0	0	0	1	0	4
W407	Lianjing4	SS200704	Lianyungang, Jiangsu	0.00	0	0	0	0.999	0	4
W408	Jindao1007	Guoshendao2004043	Dongli, Tianjin	0.00	0	0	0	0.999	0	4
W409	Huajing5	SS200505	Huaibei, Jiangsu	0.00	0	0	0	1	0	4
W410	Huajing6	SS200706	Huaibei, Jiangsu	0.00	0	0	0	1	0	4
W411	Yangfujing7	Sushendao200413	Lixiahe, Jiangsu	0.00	0	0	0	1	0	4
W412	Yangfujing8	Sushendao200608	Lixiahe, Jiangsu	0.00	0	0	0	0.999	0	4
W413	Zhendao99	SS200106	Zhenjiang, Jiangsu	0.00	0	0	0	1	0	4
W414	Nanjing42	Sushendao200616	Nanjing, Jiangsu	0.00	0	0	0	1	0	4
W415	Lianjing2	Guoshendao990021	Lianyungang, Jiangsu	0.00	0	0	0	1	0	4
W416	Huifeng1	unknown	Yancheng, Jiangsu	0.00	0	0	0	1	0	4
W417	Huifeng2	unknown	Yancheng, Jiangsu	0.00	0	0	0	1	0	4
W418	Yandao8	816922/H88-39	Yancheng, Jiangsu	0.00	0	0	0	1	0	4
W419	Wuyujing21	Sushendao200705	Wujin, Jiangsu	0.00	0	0	0	1	0	4
W420	Shashani	Heishendao2006011	Haerbin, Heilongjiang	0.00	0	0	0	1	0	4
W421	Muzhan4	Heishendao2005007	Mudanjiang, Heilongjiang	0.00	0	0	0	1	0	4
W422	Mudanjiang29	Heishendao2006007	Mudanjiang, Heilongjiang	0.00	0	0	0	1	0	4
W423	Mudanjiang28	Heishendao2006006	Mudanjiang, Heilongjiang	0.55	0	0	0	1	0	4
W424	Mudanjiang27	Heishendao2005006	Mudanjiang, Heilongjiang	0.25	0	0	0	1	0	4
W425	Kenyu20	Pin1/Jijing13	Nongken, Heilongjiang	0.00	0	0	0	1	0	4
W426	Heijing8	Heishendao2007009	Haerbin, Heilongjiang	1.08	0	0	0	1	0	4
W427	Hejiang21	Hejiang20/Puxuan10	Haerbin, Heilongjiang	0.00	0	0	0	1	0	4

W428	Beidao4	Heishendao2010006	Haerbin, Heilongjiang	0.81	0	0	0	1	0	4
W429	Beidao3	Heishendao2000002	Haerbin, Heilongjiang	0.00	0	0	0	1	0	4
W430	Suijing12	HS2009013	Suihua, Heilongjiang	0.00	0	0	0	1	0	4
W431	Songjing12	HS2008003	Songhuajiang, Heilongjiang	0.00	0	0	0	1	0	4
W432	Songjing11	HS2007006	Songhuajiang, Heilongjiang	0.00	0	0	0	0.999	0	4
W433	Songjing10	HS2005005	Songhuajiang, Heilongjiang	0.00	0	0	0	0.999	0	4
W434	Dongnong430	HS2009002	Haerbin, Heilongjiang	0.00	0	0	0	0.999	0	4
W435	Dongnong424	HS2005002	Haerbin, Heilongjiang	0.00	0	0	0	0.999	0	4
W436	Longnuo3	HS2009015	Haerbin, Heilongjiang	0.00	0	0	0	0.999	0	4
W437	Longjing28	HS2009011	Haerbin, Heilongjiang	0.00	0	0	0	0.999	0	4
W438	Longjing27	HS2009010	Haerbin, Heilongjiang	0.00	0	0	0	1	0	4
W439	Longjing26	HS2009008	Haerbin, Heilongjiang	0.00	0	0	0	0.999	0	4
W440	Longjing25	HS2009009	Haerbin, Heilongjiang	0.00	0	0	0	0.999	0	4
W441	Longjing24	HS2008017	Haerbin, Heilongjiang	0.00	0	0	0	1	0	4
W442	Longjing22	HS2008010	Haerbin, Heilongjiang	0.00	0	0	0	1	0	4
W443	Longjing21	HS2008008	Haerbin, Heilongjiang	0.00	0	0	0	0.999	0	4
W444	Longjing20	HS2007004	Haerbin, Heilongjiang	0.00	0	0	0	0.999	0	4
W445	Longjing19	HS2007003	Haerbin, Heilongjiang	0.00	0	0	0	1	0	4
W446	Longjing18	HS2007002	Haerbin, Heilongjiang	0.00	0.003	0.002	0.098	0.873	0.025	4
W447	Longjing17	HS2007001	Haerbin, Heilongjiang	0.00	0	0	0	0.999	0	4
W448	Longjing16	HS2006002	Haerbin, Heilongjiang	0.00	0	0	0	0.999	0	4
W449	Longjing15	HS2006001	Haerbin, Heilongjiang	0.00	0	0	0	0.999	0	4
W450	Zhonglongdao1	HS2008004	Haerbin, Heilongjiang	0.00	0	0	0	0.999	0	4
W451	Longdao8	Muzhan3/Dongqin241	Haerbin, Heilongjiang	0.00	0	0	0	1	0	4
W452	Longdao6	HS2006003	Haerbin, Heilongjiang	0.00	0	0	0	1	0	4
W453	Longdao5	HS2006002	Haerbin, Heilongjiang	0.00	0	0	0	0.999	0	4
W454	Longdao4	HS2005003	Haerbin, Heilongjiang	0.00	0	0	0	0.999	0	4
W455	Kendao19	HS2009012	Haerbin, Heilongjiang	0.00	0	0	0	0.999	0	4
W456	Kendao18	HS2008012	Haerbin, Heilongjiang	0.00	0	0	0	0.999	0	4

W457	Kendao12	HS2006009	Haerbin, Heilongjiang	0.00	0.003	0.003	0.08	0.866	0.049	4
W458	Kendao13	HS2008011	Haerbin, Heilongjiang	0.00	0	0	0	0.999	0	4
W459	Kendao20	HS2009003	Haerbin, Heilongjiang	0.00	0	0	0	0.999	0	4
W460	Longdun106	HS2008016	Haerbin, Heilongjiang	0.00	0	0	0	0.999	0	4
W461	Longdun105	HS2007008	Haerbin, Heilongjiang	0.00	0	0	0	0.999	0	4
W462	Sanjiang2	HS2008018	Haerbin, Heilongjiang	0.00	0	0	0	0.999	0	4
W463	Nongxiang20	Xiangshendao2010038	Changsha, Hunan	0.24	0.861	0	0.001	0.138	0	1
W464	Nongxiangyou205	Xiangshendao2010029	Changsha, Hunan	0.62	0.999	0	0	0	0	1
W465	Nongxiangyou206	NongxiangA/R206	Changsha, Hunan	0.26	0.999	0	0	0	0	1
W466	Yuzhenxiang	XS2008038	Changsha, Hunan	0.25	0.999	0	0	0	0	1
W467	Xiangwanxian17	XS2008035	Changsha, Hunan	0.00	0.999	0	0	0	0	1
W468	Huanghuazhan	ES2007017	Changsha, Hunan	0.00	0.998	0	0	0	0.001	1
W469	Nongxiang18	Xiangshendao2010038	Changsha, Hunan	0.25	1	0	0	0	0	1
W470	Ribenqing	Yamabiko/Xingfeng	Haerbin, Heilongjiang	0.00	1	0	0	0	0	1
W471	Tijin	NL150	Haerbin, Heilongjiang	0.00	0.999	0	0	0	0	1
W472	M1004	Oochikara Mutant	Haerbin, Heilongjiang	0.00	1	0	0	0	0	1
W473	Zhongguo91	NL274	Haerbin, Heilongjiang	0.00	1	0	0	0	0	1
W474	Qiutainxiaoding	Yueguang/Aoyu292	Haerbin, Heilongjiang	0.00	1	0	0	0	0	1
W475	Cai	Daobei43/Beiming	Haerbin, Heilongjiang	0.00	1	0	0	0	0	1
W476	Qiuguang	Liming/fengjin	Haerbin, Heilongjiang	0.00	1	0	0	0	0	1
W477	Kangbingyueguang	Nonglin22/Nonglin1-R	Haerbin, Heilongjiang	0.00	1	0	0	0	0	1
W478	Youzhiyueguang	Nonglin22/Nonglin1-H	Haerbin, Heilongjiang	0.00	1	0	0	0	0	1
W479	Kasalath	Introduced from Japan	Haerbin, Heilongjiang	0.79	0.999	0	0	0	0	1
W480	Xiangchuanwuxinbaimi	unknown	Haerbin, Heilongjiang	1.09	0.999	0	0	0	0	1
W481	Wuyujing24	Sushendao201209	Hongze, Jiangsu	0.00	1	0	0	0	0	1
W482	Wuyujing27	Sushendao201209	Hongze, Jiangsu	0.00	1	0	0	0	0	1
W483	Heimixiandao-1	09-02543	Nanjing, Jiangsu	0.00	1	0	0	0	0	1
W484	Heimijingdao-2	09-02544	Nanjing, Jiangsu	0.00	1	0	0	0	0	1
W485	Zidao	unknown	Nanjing, Jiangsu	0.36	1	0	0	0	0	1
W486	9311	GS2001002	Yangzhou, Jiangsu	0.00	1	0	0	0	0	1
W487	Zacaodao	unknown	Nanjing, Jiangsu	0.00	1	0	0	0	0	1

W488	Youmang429	unknown	Nanjing, Jiangsu	0.00	1	0	0	0	0	1
W489	Wanqu429	unknown	Nanjing, Jiangsu	0.00	1	0	0	0	0	1
W490	Zhili429	unknown	Nanjing, Jiangsu	0.00	1	0	0	0	0	1
W491	Si4029	Introduction 4029	Sihong, Jiangsu	0.00	1	0	0	0	0	1
W492	Si4031	Introduction4031	Sihong, Jiangsu	0.00	1	0	0	0	0	1
W493	Si4033	Introduction4033	Sihong, Jiangsu	0.00	1	0	0	0	0	1
W494	Si4039	Introduction4039	Sihong, Jiangsu	0.00	1	0	0	0	0	1
W495	Si4040	Introduction4040	Sihong, Jiangsu	0.00	1	0	0	0	0	1
W496	Si4041	Introduction4041	Sihong, Jiangsu	0.00	1	0	0	0	0	1
W497	Si4049	Introduction4049	Sihong, Jiangsu	0.00	1	0	0	0	0	1
W498	Si4079	Introduction4079	Sihong, Jiangsu	0.00	1	0	0	0	0	1
W499	Si4081	Introduction4081	Sihong, Jiangsu	0.25	1	0	0	0	0	1
W500	Si4082	Introduction4082	Sihong, Jiangsu	0.00	1	0	0	0	0	1
W501	Si4139	Introduction4139	Sihong, Jiangsu	0.00	1	0	0	0	0	1
W502	Si4152	Introduction4152	Sihong, Jiangsu	0.00	1	0	0	0	0	1
W503	Si4161	Introduction4161	Sihong, Jiangsu	0.00	1	0	0	0	0	1
W504	Si4229	Introduction4229	Sihong, Jiangsu	0.00	1	0	0	0	0	1
W505	Si4230	Introduction4230	Sihong, Jiangsu	0.00	1	0	0	0	0	1
W506	Si4251	Introduction4251	Sihong, Jiangsu	0.00	1	0	0	0	0	1
W507	Si4252	Introduction4252	Sihong, Jiangsu	0.00	1	0	0	0	0	1
W508	Si4259	Introduction4259	Sihong, Jiangsu	0.00	1	0	0	0	0	1
W509	Si4263	Introduction4263	Sihong, Jiangsu	0.34	1	0	0	0	0	1
W510	Si4280	Introduction4280	Sihong, Jiangsu	0.00	1	0	0	0	0	1
W511	Si4330	Introduction4330	Sihong, Jiangsu	0.00	0.999	0	0	0	0	1
W512	Si4360	Introduction4360	Sihong, Jiangsu	0.00	1	0	0	0	0	1
W513	Si4364	Introduction4364	Sihong, Jiangsu	0.00	1	0	0	0	0	1
W514	Digudao	Mutant	Sihong, Jiangsu	0.00	1	0	0	0	0	1
W515	Si4385	Introduction4385	Sihong, Jiangsu	0.00	1	0	0	0	0	1
W516	Si4386	Introduction4386	Sihong, Jiangsu	1.36	0.999	0	0	0	0	1
W517	Sihao4141	Sidao8/zhongdan2	Sihong, Jiangsu	0.00	1	0	0	0	0	1
W518	Ningjinghui096	Introduction 096	Nanjing, Jiangsu	0.00	1	0	0	0	0	1

W519	Ningjinghui117	Introduction117	Nanjing, Jiangsu	0.00	1	0	0	0	0	1
W520	Ningjinghui145	Introduction145	Nanjing, Jiangsu	0.26	1	0	0	0	0	1
W521	Ningjinghui166	Introduction166	Nanjing, Jiangsu	0.00	1	0	0	0	0	1
W522	Ningjinghui208	Introduction208	Nanjing, Jiangsu	0.00	0.999	0	0	0	0	1
W523	Ningjinghui210	Introduction210	Nanjing, Jiangsu	0.00	1	0	0	0	0	1
W524	Ningjinghui237	Introduction237	Nanjing, Jiangsu	0.00	1	0	0	0	0	1
W525	Ningjinghui246	Introduction246	Nanjing, Jiangsu	0.00	1	0	0	0	0	1
W526	Ningjinghui260	Introduction260	Nanjing, Jiangsu	0.19	1	0	0	0	0	1
W527	Ningjinghui285	Introduction285	Nanjing, Jiangsu	0.00	1	0	0	0	0	1
W528	Ningjinghui286	Introduction286	Nanjing, Jiangsu	0.25	1	0	0	0	0	1
W529	Ningjinghui290	Introduction290	Nanjing, Jiangsu	0.29	1	0	0	0	0	1
W530	Ningjinghui292	Introduction292	Nanjing, Jiangsu	0.00	1	0	0	0	0	1
W531	Ningjinghui293	Introduction293	Nanjing, Jiangsu	0.27	1	0	0	0	0	1
W532	Ningjinghui296	Introduction296	Nanjing, Jiangsu	0.29	1	0	0	0	0	1
W533	Ningjinghui298	Introduction298	Nanjing, Jiangsu	0.00	1	0	0	0	0	1
W534	Ningjinghui338	Introduction338	Nanjing, Jiangsu	0.00	1	0	0	0	0	1
W535	Hongyin1009	Introduction1009	Nanjing, Jiangsu	0.00	1	0	0	0	0	1
W536	Hongyin1010	Introduction1010	Nanjing, Jiangsu	0.00	1	0	0	0	0	1
W537	Hongyin1011	Introduction1011	Nanjing, Jiangsu	0.00	1	0	0	0	0	1
W538	Hongyin1012	Introduction1012	Nanjing, Jiangsu	0.00	1	0	0	0	0	1
W539	Cai 1	Yongxi8721	Haerbin, Heilongjiang	0.00	1	0	0	0	0	1
W540	Nannongjing1R	C57/IR28//Jingyin37	Nanjing, Jiangsu	0.00	1	0	0	0	0	1
W541	Nannongjing2R	Ke6/Ke3//Jingyin35	Nanjing, Jiangsu	0.00	1	0	0	0	0	1
W542	Nannongjing3R	Zhanli/Jinghui7623	Nanjing, Jiangsu	0.00	1	0	0	0	0	1
W543	Ningjing1R-34	Ningjing1/R254// Ningjing1-CSSL34	Nanjing, Jiangsu	0.00	0.494	0.227	0.009	0.149	0.122	1
