# Supplementary material for: Screening germplasm and detecting QTLs for mesocotyl elongation trait in rice (Oryza sativa L.) by association mapping
Source: BMC Genom Data. 2023 Feb 15;24:8. doi: 10.1186/s12863-023-01107-8 (PMC9930352; doi:10.1186/s12863-023-01107-8)
Supplement: Supplementary file 3 — Additional file 3: Table S3. Summary statistics for the 262 SSR markers used in this study. [file 12863_2023_1107_MOESM3_ESM.pdf]

Additional file 3: Table S3. Summary statistics for the 262 SSR markers used in this study

Code	Locus	Chr.	Position (cM)	Allele number	Gene diversity	PIC	Code	Locus	Chr. No.	Position (cM)	Allele number	Gene diversity	PIC
1	RM84	1	18.8	7	0.4553	0.4234	133	RM136	6	53	18	0.8589	0.8456
2	RM1-003	1	19.9	12	0.8144	0.7910	134	RM3330	6	61.6	14	0.8807	0.8704
3	RM283	1	25.4	13	0.8542	0.8406	135	RM3187	6	73.2	6	0.6694	0.6115
4	RM3453	1	25.4	10	0.7073	0.6754	136	RM7579	6	84.5	6	0.7437	0.6967
5	RM1	1	29.7	20	0.8680	0.8555	137	RM8239	6	91.9	5	0.7374	0.6936
6	RM259	1	38.8	6	0.7023	0.6481	138	RM454	6	99.3	4	0.5585	0.4976
7	RM583	1	43.2	5	0.2928	0.2775	139	RM7309	6	100.3	15	0.8688	0.8567
8	RM490	1	51	9	0.8182	0.7936	140	RM528	6	100.8	11	0.7281	0.6933
9	RM8095	1	60.6	8	0.3165	0.3038	141	RM3138	6	110.6	12	0.8310	0.8114
10	RM140	1	65.4	8	0.3165	0.3038	142	RM162	6	114.9	20	0.9002	0.8925
11	RM562	1	78.4	21	0.9205	0.9149	143	RM6811	6	115.6	15	0.8976	0.8888
12	RM9	1	92.4	16	0.8110	0.7907	144	RM345	6	123.9	7	0.5865	0.5352
13	RM129	1	93	5	0.3513	0.3376	145	RM5753	6	124.4	18	0.8819	0.8729
14	RM5	1	98.5	9	0.8229	0.7989	146	RM295	7	0	13	0.7155	0.6871
15	RM1231	1	123.2	15	0.9002	0.8921	147	RM125	7	24.8	4	0.5714	0.4771
16	RM128	1	126.5	14	0.8280	0.8093	148	RM180	7	30.1	8	0.6577	0.6216
17	RM297	1	132	10	0.7689	0.7359	149	RM542	7	34.7	7	0.8091	0.7808
18	RM246	1	134.6	16	0.8663	0.8522	150	RM8263	7	35.7	8	0.6852	0.6330
19	RM212	1	135.8	5	0.4140	0.3925	151	RM418	7	42.1	9	0.7152	0.6723
20	RM5389	1	142.4	12	0.8520	0.8350	152	RM346	7	47	9	0.7845	0.7569
21	RM486	1	153.5	10	0.7213	0.6804	153	RM2530	7	53.4	14	0.8644	0.8510
22	RM265	1	155.9	7	0.6916	0.6526	154	RM336	7	61	12	0.8218	0.8037
23	RM3482	1	157.6	13	0.8211	0.8056	155	RM5380	7	67	9	0.7775	0.7418
24	RM6831	1	181.8	8	0.7594	0.7258	156	RM6011	7	73.2	12	0.8875	0.8773
25	RM14	1	194	14	0.8702	0.8580	157	RM505	7	78.6	8	0.8288	0.8076

26	RM5340	2	36.3	15	0.8666	0.8533	158	RM3589	7	89.8	13	0.8317	0.8119
27	RM7288	2	42.4	22	0.8965	0.8885	159	RM11	7	93.8	8	0.7675	0.7345
28	RM5356	2	43.3	10	0.8475	0.8291	160	RM234	7	93.9	13	0.7939	0.7738
29	RM1358	2	48.1	10	0.8298	0.8080	161	RM134	7	99.6	10	0.8353	0.8155
30	RM1313	2	51.1	8	0.7806	0.7470	162	RM1306	7	116.1	13	0.8256	0.8112
31	RM324	2	51.1	5	0.3868	0.3520	163	RM82	7	128.9	4	0.3191	0.3008
32	RM327	2	51.9	10	0.8291	0.8073	164	RM506	8	0	12	0.8167	0.7951
33	RM301	2	53.5	7	0.7750	0.7446	165	RM1019	8	0.5	17	0.8729	0.8605
34	RM300	2	54.6	15	0.8814	0.8701	166	RM152	8	9.4	11	0.7957	0.7692
35	RM262	2	70.2	9	0.7740	0.7415	167	RM1235	8	12.8	5	0.5953	0.5205
36	RM5427	2	84.6	11	0.7011	0.6630	168	RM6863	8	16.4	10	0.7709	0.7351
37	RM3688	2	88.2	8	0.8450	0.8260	169	RM4085	8	35.7	12	0.8416	0.8252
38	RM183	2	93.5	15	0.8638	0.8496	170	RM544	8	38.5	15	0.9124	0.9058
39	RM5804	2	98.2	7	0.7802	0.7479	171	RM8243	8	50.8	10	0.8716	0.8580
40	RM106	2	101.5	18	0.8988	0.8907	172	RM25	8	52.2	12	0.7782	0.7514
41	RM6361	2	102.9	8	0.7338	0.6977	173	RM331	8	59	10	0.8044	0.7770
42	RM573	2	118.1	14	0.8291	0.8103	174	RM72	8	60.9	14	0.8206	0.8025
43	RM450	2	122.8	7	0.7993	0.7720	175	RM6215	8	66.8	10	0.7603	0.7329
44	RM7598	2	126.4	7	0.5601	0.5400	176	RM7556	8	86.7	11	0.8488	0.8304
45	RM263	2	127.5	13	0.8686	0.8552	177	RM6976	8	92.2	19	0.9186	0.9128
46	RM112	2	137.5	8	0.6756	0.6466	178	RM80	8	103.7	12	0.6538	0.6348
47	RM525	2	143.7	19	0.8469	0.8337	179	RM502	8	109.3	15	0.8647	0.8511
48	RM213	2	150.5	10	0.8415	0.8226	180	RM3754	8	112.6	7	0.8278	0.8053
49	RM208	2	154.1	6	0.6617	0.6053	181	RM6948	8	114.4	7	0.7177	0.6836
50	RM3850	2	156.3	12	0.8369	0.8186	182	RM433	8	116	6	0.2740	0.2650
51	RM498	2	156.3	15	0.8154	0.7949	183	RM281	8	128.1	14	0.8366	0.8174
52	RM48	2	191.2	10	0.4634	0.4476	184	RM264	8	138.2	12	0.8705	0.8568

53	RM266	2	192.2	11	0.7590	0.7319	185	RM1328	9	0	12	0.8697	0.8558
54	RM535	2	195.7	18	0.8622	0.8502	186	RM8206	9	3.2	11	0.7651	0.7364
55	RM132	3	3.9	4	0.4209	0.3874	187	RM524	9	42.5	10	0.8289	0.8080
56	RM1332	3	11.5	4	0.6755	0.6117	188	RM3912	9	46.3	10	0.8468	0.8274
57	RM5849	3	18.4	15	0.8129	0.7919	189	RM566	9	50.7	9	0.7447	0.7090
58	RM489	3	20.3	13	0.8845	0.8733	190	RM434	9	57.7	9	0.8556	0.8396
59	RM545	3	24.7	5	0.6003	0.5413	191	RM3600	9	62.7	12	0.8003	0.7764
60	RM5480	3	25.9	11	0.6131	0.5872	192	RM24481	9	63	19	0.8919	0.8834
61	RM3467	3	28.2	14	0.8883	0.8782	193	RM3533	9	65.1	13	0.8226	0.8013
62	RM3766	3	34.8	10	0.7372	0.7071	194	RM6570	9	68.2	9	0.5562	0.5314
63	RM7	3	36.9	6	0.7142	0.6641	195	RM410	9	79.3	9	0.7164	0.6764
64	RM5639	3	39.8	8	0.7246	0.6812	196	RM257	9	79.7	11	0.7693	0.7489
65	RM7197	3	44.4	11	0.8202	0.7965	197	RM201	9	81.2	9	0.7668	0.7335
66	RM7345	3	48.8	7	0.6759	0.6398	198	OSR28	9	85.4	12	0.8476	0.8309
67	RM282	3	55.8	11	0.8506	0.8331	199	RM5384	9	90.7	10	0.8647	0.8499
68	RM338	3	61.9	8	0.7514	0.7152	200	RM1013	9	93.5	7	0.7656	0.7324
69	RM218	3	67.8	9	0.7234	0.6939	201	RM7492	10	0	12	0.8680	0.8542
70	RM232	3	76.7	12	0.8337	0.8154	202	RM7545	10	7.6	25	0.9429	0.9400
71	RM7403	3	82.3	4	0.2100	0.1954	203	RM6646	10	13.3	9	0.8229	0.8038
72	RM6266	3	94.9	6	0.6604	0.6168	204	RM244	10	15	6	0.4791	0.4428
73	RM7097	3	115.6	7	0.7956	0.7653	205	RM216	10	24.8	3	0.5886	0.5139
74	RM135	3	120.4	9	0.7836	0.7538	206	RM311	10	25.2	8	0.7991	0.7730
75	RM168	3	122.8	12	0.5825	0.5580	207	RM184	10	41.6	5	0.7575	0.7166
76	RM186	3	127.4	7	0.7779	0.7416	208	RM1125	10	46.8	14	0.8451	0.8307
77	RM16	3	131.5	6	0.3004	0.2792	209	RM258	10	48.8	8	0.6815	0.6146
78	RM5475	3	137.9	21	0.9146	0.9084	210	RM5629	10	53.6	11	0.7945	0.7705
79	RM416	3	140.1	9	0.7255	0.6908	211	RM6100	10	53.9	5	0.2962	0.2776

80	RM6712	3	158.2	11	0.7810	0.7579	212	RM1108	10	55.3	6	0.3436	0.3266
81	RM448	3	191.6	12	0.8680	0.8545	213	RM3773	10	58.9	17	0.9025	0.8945
82	RM148	3	249.3	8	0.7364	0.6915	214	RM269	10	69.6	5	0.6568	0.5924
83	RM307	4	0	11	0.8399	0.8207	215	RM5352	10	71.4	5	0.5214	0.4307
84	RM335	4	5.4	14	0.8281	0.8068	216	RM304	10	73	6	0.1524	0.1501
85	RM518	4	7.9	9	0.8071	0.7804	217	RM171	10	73	5	0.6373	0.5680
86	RM3471	4	16.7	17	0.8944	0.8859	218	RM6160	10	81	8	0.5780	0.5424
87	RM4835	4	18.3	10	0.5937	0.5173	219	RM590	10	83.3	9	0.8181	0.7938
88	RM5687	4	25.4	9	0.7632	0.7362	220	RM333	10	110.4	7	0.3435	0.3324
89	RM6314	4	41.5	14	0.8352	0.8173	221	RM286	11	0.1	8	0.6986	0.6675
90	RM471	4	53.8	10	0.7679	0.7449	222	RM6327	11	1.7	20	0.8905	0.8809
91	RM5951	4	56.1	5	0.7103	0.6577	223	RM1240	11	6.5	15	0.8672	0.8545
92	RM142	4	60.2	16	0.8788	0.8682	224	RM7557	11	9.2	5	0.5908	0.5381
93	RM6997	4	62.1	12	0.7765	0.7502	225	RM1812	11	10.3	10	0.6125	0.5573
94	RM7563	4	68.3	8	0.8382	0.8188	226	RM6544	11	19.8	5	0.4537	0.4237
95	RM6114	4	72	11	0.8520	0.8363	227	RM3133	11	32.7	5	0.6913	0.6371
96	RM6589	4	85.2	8	0.8290	0.8068	228	RM167	11	37.5	4	0.6561	0.5853
97	RM317	4	96	6	0.7120	0.6633	229	RM3701	11	45.3	10	0.7408	0.7032
98	RM6089	4	97.7	10	0.8196	0.7969	230	RM7391	11	54.3	9	0.8083	0.7872
99	RM3513	4	99.6	8	0.5704	0.5274	231	RM7303	11	64.2	4	0.0857	0.0844
100	RM3836	4	108.2	11	0.8218	0.8002	232	RM7120	11	66.6	8	0.7537	0.7207
101	RM280	4	128.9	3	0.4034	0.3477	233	RM287	11	68.6	8	0.5740	0.5343
102	RM559	4	129.6	6	0.7127	0.6693	234	RM457	11	78.8	8	0.6810	0.6353
103	RM349	4	146.8	8	0.7201	0.6866	235	RM5349	11	79.1	6	0.4049	0.3681
104	RM348	4	160.8	4	0.5049	0.4535	236	RM209	11	84.7	11	0.7480	0.7084
105	RM1182	5	3	11	0.8521	0.8350	237	RM21	11	85.7	12	0.8827	0.8710
106	RM153	5	3	13	0.8393	0.8225	238	RM7170	11	101.9	15	0.8600	0.8454

107	RM122	5	3	6	0.2274	0.2154	239	RM206	11	102.9	2	0.0802	0.0770
108	RM159	5	5.4	14	0.8912	0.8812	240	RM7163	11	112.4	6	0.6716	0.6068
109	RM267	5	25	12	0.8472	0.8345	241	RM6293	11	117.3	14	0.8852	0.8743
110	RM437	5	31.5	2	0.2544	0.2220	242	RM224	11	120.1	13	0.8163	0.7928
111	RM3193	5	36.4	6	0.7488	0.7171	243	RM20	12	3.2	15	0.8560	0.8401
112	RM574	5	41	7	0.7881	0.7633	244	RM19	12	20.9	11	0.8114	0.7884
113	RM249	5	50.2	7	0.7284	0.6844	245	RM247	12	26.7	5	0.5642	0.5265
114	RM6082	5	53.5	14	0.8462	0.8326	246	RM6296	12	26.7	3	0.4940	0.4048
115	RM598	5	62.7	6	0.5606	0.5018	247	RM7619	12	38.1	5	0.3330	0.2992
116	RM473B	5	78.7	10	0.8597	0.8438	248	RM512	12	39.4	15	0.8544	0.8415
117	RM164	5	91.4	8	0.6603	0.6275	249	RM5746	12	39.4	3	0.3856	0.3175
118	RM188	5	95.3	11	0.8382	0.8199	250	RM277	12	48.2	9	0.6972	0.6518
119	RM161	5	96.9	17	0.8978	0.8889	251	RM1337	12	51.5	10	0.8187	0.7962
120	RM305	5	96.9	7	0.5860	0.5286	252	RM511	12	59.8	11	0.7115	0.6851
121	RM3170	5	115.4	14	0.8806	0.8686	253	RM1246	12	65.3	5	0.5856	0.5221
122	RM480	5	130.6	18	0.8665	0.8552	254	RM7102	12	71.8	10	0.7793	0.7542
123	RM5818	5	144.9	11	0.8239	0.8032	255	RM309	12	73	9	0.7966	0.7692
124	RM8109	6	1.7	10	0.7823	0.7525	256	RM6869	12	75.8	15	0.8445	0.8285
125	RM508	6	2.3	11	0.8319	0.8134	257	RM463	12	75.5	8	0.4614	0.4383
126	RM510	6	11.5	12	0.8346	0.8194	258	RM3331	12	89.5	9	0.7118	0.6650
127	RM225	6	26.2	12	0.7592	0.7256	259	RM270	12	91.3	9	0.7443	0.7070
128	RM405	6	28.6	6	0.7190	0.6705	260	RM5479	12	95.4	18	0.7547	0.7340
129	RM2126	6	32.7	11	0.7279	0.6886	261	RM17	12	107.4	10	0.8396	0.8223
130	RM50	6	32.7	7	0.6559	0.6250	262	RM12	12	107.4	4	0.5466	0.4616
131	RM276	6	33.5	13	0.8538	0.8374		Total Alleles			2649		
132	RM314	6	33.6	8	0.7761	0.7435		Mean			10.107	0.734	0.706
